# High-density lipoproteins suppress Aβ-induced PBMC adhesion to human endothelial cells in bioengineered vessels and in monoculture

**DOI:** 10.1186/s13024-017-0201-0

**Published:** 2017-08-22

**Authors:** Jérôme Robert, Emily B. Button, Sophie Stukas, Guilaine K. Boyce, Ebrima Gibbs, Catherine M. Cowan, Megan Gilmour, Wai Hang Cheng, Sonja K. Soo, Brian Yuen, Arvin Bahrabadi, Kevin Kang, Iva Kulic, Gordon Francis, Neil Cashman, Cheryl L. Wellington

**Affiliations:** 1 Department of Pathology and Laboratory Medicine, University of British Columbia, Vancouver, BC V6T 1Z3 Canada; 2 Djavad Mowafaghian Centre for Brain Health, University of British Columbia, 2215 Wesbrook Mall, Vancouver, BC V6T 1Z3 Canada; 3 Department of Neurology, University of British Columbia, Vancouver, BC V6T 2B5 Canada; 4 Department of Zoology, University of British Columbia, Vancouver, BC V6T 2B5 Canada; 5 Department of Medicine, University of British Columbia, Vancouver, BC V6Z 1Y6 Canada

**Keywords:** Alzheimer’s disease, Beta-amyloid, High-density lipoprotein, Engineered vessel, Endothelial cells

## Abstract

**Background:**

Alzheimer’s Disease (AD), characterized by accumulation of beta-amyloid (Aβ) plaques in the brain, can be caused by age-related failures to clear Aβ from the brain through pathways that involve the cerebrovasculature. Vascular risk factors are known to increase AD risk, but less is known about potential protective factors. We hypothesize that high-density lipoproteins (HDL) may protect against AD, as HDL have vasoprotective properties that are well described for peripheral vessels. Epidemiological studies suggest that HDL is associated with reduced AD risk, and animal model studies support a beneficial role for HDL in selectively reducing cerebrovascular amyloid deposition and neuroinflammation. However, the mechanism by which HDL may protect the cerebrovascular endothelium in the context of AD is not understood.

**Methods:**

We used peripheral blood mononuclear cell adhesion assays in both a highly novel three dimensional (3D) biomimetic model of the human vasculature composed of primary human endothelial cells (EC) and smooth muscle cells cultured under flow conditions, as well as in monolayer cultures of ECs, to study how HDL protects ECs from the detrimental effects of Aβ.

**Results:**

Following Aβ addition to the abluminal (brain) side of the vessel, we demonstrate that HDL circulated within the lumen attenuates monocyte adhesion to ECs in this biofidelic vascular model. The mechanism by which HDL suppresses Aβ-mediated monocyte adhesion to ECs was investigated using monotypic EC cultures. We show that HDL reduces Aβ-induced PBMC adhesion to ECs independent of nitric oxide (NO) production, miR-233 and changes in adhesion molecule expression. Rather, HDL acts through scavenger receptor (SR)-BI to block Aβ uptake into ECs and, in cell-free assays, can maintain Aβ in a soluble state. We confirm the role of SR-BI in our bioengineered human vessel.

**Conclusion:**

Our results define a novel activity of HDL that suppresses Aβ-mediated monocyte adhesion to the cerebrovascular endothelium.

**Electronic supplementary material:**

The online version of this article (doi:10.1186/s13024-017-0201-0) contains supplementary material, which is available to authorized users.

## Background

Alzheimer’s disease (AD) is the leading cause of dementia with over 44 million affected persons worldwide and has no effective treatment [1]. AD is believed to arise from the toxic effects of amyloidogenic beta-amyloid peptides (Aβ), which form higher order structures including oligomers and fibrils that accumulate as amyloid plaques in the brain parenchyma and cerebral vessels [2]. The role of the cerebrovasculature in AD has gained increased recognition in the last decade, as AD risk is increased by vascular risk factors, including hypertension, type 2 diabetes mellitus (T2DM), and hypercholesterolemia [3–5]. Most AD autopsy cases have evidence of cerebral small vessel disease, including cerebral amyloid angiopathy (CAA) and microvascular degeneration, with micro- and macro-infarcts reported in over 80% of very old AD subjects [6]. The cerebrovasculature plays a pivotal role in clearing Aβ from the brain [7], and activation of cerebrovascular endothelial cells (ECs) has been reported in AD brain [8].

High-density lipoproteins (HDL) are a heterogeneous mixture of circulating lipoprotein particles that are composed of, in addition to apoA-I, cholesterol and phospholipids, over 200 lipid species and at least 95 proteins in normolipidemic plasma [9, 10]. Intriguingly, the elevated concentration of plasma high-density lipoprotein cholesterol (HDL-C), which is associated with reduced cardiovascular disease risk [11], is also negatively correlated with cognitive decline [12]. Preclinical studies also support a beneficial role of HDL on the cerebrovasculature. Specifically, transgenic overexpression of human apoA-I, the major protein component on HDL, from its endogenous promoter in the liver and intestine, reduces neuroinflammation, improves cognitive function and selectively reduces cerebrovascular amyloid in the APP/PS1 model of amyloidosis, whereas deletion of apoA-I, the major protein component of HDL, has the opposite effects [13, 14]. We recently reported that a single intravenous injection of reconstituted HDL reduces soluble brain Aβ40 and Aβ42 levels after 24 h in symptomatic APP/PS1 mice [15].

Systemically circulating HDL may therefore benefit the brain, however, the mechanisms by which it may do so are unknown. HDL is well established to possess several potent vasoprotective functions in peripheral vascular endothelial cells (ECs) including reducing inflammation, increasing vascular tone through promoting endothelial nitric oxide (NO) synthase activity, and suppressing vascular adhesion molecule expression [16]. Using peripheral blood mononuclear cell (PBMC) adherence assays, here we show that Aβ induces PMBC adhesion, a classical measure of EC activation, in cerebral microvascular ECs through a mechanism distinct from classical inflammatory stimulation by tumor necrosis factor-alpha (TNF-α). We also demonstrate that HDL robustly suppresses Aβ-induced monocyte adhesion to ECs both in monolayer cultures as well as in a novel 3 dimensional (3D) tissue engineered human vessel.

## Methods

### Fabrication of tissue engineered vessels

Scaffold-directed human engineered vessels were generated under flow bioreactor conditions as described [17]. Briefly, non-woven polyglycolic-acid meshes (PGA; BMS) were coated with polycoprolactone (PCL, Purac) and polyglycolic acid (PLA, Purac) by dipping into a 1.75% (*w*/w/w) solution of PCL/PLA/tetrahydrofuran (Sigma Aldrich). A tubular shape (length 10 mm and inner diameter 2 mm) was obtained by heat welding before external coating with a 10% (*w*/w) PCL/tetrahydrofuran solution. After sterilization with 70% ethanol for 30 min followed by 3 washes with PBS, scaffolds were pre-incubated in advanced DMEM overnight before cell seeding. Human umbilical cord smooth muscle cells were isolated as described [17] and 2 × 10^6^ cells/cm^2^ were seeded in the inner surface of the vascular scaffold using fibrinogen (10 mg/mL clottable protein, Sigma Aldrich) and thrombin (10 mU) as cell carriers [18]. After a short static incubation period of 3 days, vascular constructs were exposed to dynamic conditioning in a flow bioreactor, where the flow of nutrient medium (Advanced DMEM (Invitrogen) with 10% FBS; 0.05% Penicillin/Streptomycin, 1% L-glutamine and 1.5 mM L-ascorbic acid) was directed through the inner lumen of the bioreactor circulation loop at 6 mL/min. After 14 days of flow conditioning, vascular grafts were endothelialzed with HUVECs (1.5 × 10^6^ cells/cm^2^) seeded into the lumen followed by cultivation under static conditions for 5 days in complete endothelial growth medium (EBM-2; Lonza) supplemented with 10% foetal bovine serum (FBS) and growth supplements to form EGM complete media FBS. After the static phase, vascular grafts were placed back in the bioreactor for 14 additional days with increasing medium flow (4 to 6 mL/min) in complete EGM-2 with 10% FBS.

### Characterization of engineered vascular tissue

For histological characterization, engineered vessels were fixed in formalin (Fisher), dehydrated through a graded ethanol series using a Sakura Tissue Processor (Sakura), embedded in paraffin and sectioned at 7 μm thickness. Sections were deparaffinised, rehydrated through a graded ethanol series and stained using haematoxylin & eosin (SigmaAldrich) following the manufacture’s instructions. For immunofluorescence analyses, tissues were cryopreserved in Cryomatrix (ThermoFisher) and sectioned at 20 μm thickness using a cryotome (Leica). Immunostaining was performed as described [17] using antibodies specific for the EC marker CD31 (clone JC/70A Biolegend, 1:50) and α-smooth muscle actin (clone 1A4 SigmaAldrich, 1:200). Sections were counter-stained with DAPI and imaged with an inverted microscope (Carl Zeiss). Endothelium barrier integrity was analyzed by injecting Evans blue (Sigma Aldrich) at a final concentration of 0.5% in the circulation loop of the bioreactor for 10 min followed by continuous PBS washing for 20 min. Vessels were cut open longitudinally and *en face* preparations were analysed macroscopically with photo documentation.

### Preparation of HDL and PBMCs

All experiments were conducted under an approved clinical protocol (UBC Clinical Ethics Research Board H14–03357). Upon receipt of written informed consent, 100 mL of fasted blood was collected from normolipidemic healthy donors into vacutainer tubes. Plasma HDL (1.063–1.21 g/mL) was isolated by sequential potassium bromide gradient ultracentrifugation as described [19]. The purity of the HDL preparations was verified by sodium dodecyl sulphate-polyacrylamide gel electrophoresis (SDS-PAGE) followed by Coomassie blue staining to ensure no low-density lipoprotein (LDL) or albumin contamination. Eight independent donors were used across the experiments, 6 isolated in-house and 2 commercially obtained (Leebioscience). Human-derived, lipid free apoA-I was a kind gift from CSL-Behring. Immortalized human THP1 monocytes (ATCC) were cultured in RPMI containing 10% FBS, 1% Pen/Strep, 2 mM L-glutamine and 0.1% β-mercaptoethanol. Primary human PBMC were isolated from healthy donors by centrifugation on a continuous density gradient (Lymphoprep™, Stemcell) following the manufacturer’s instructions. Freshly isolated PBMC were fluorescently labeled with 10 μM of Cell-Tracker Red for 30 min (Invitrogen) following the manufacturer’s recommendations.

### Monocyte adhesion in engineered vessels

**Fig. 1:**
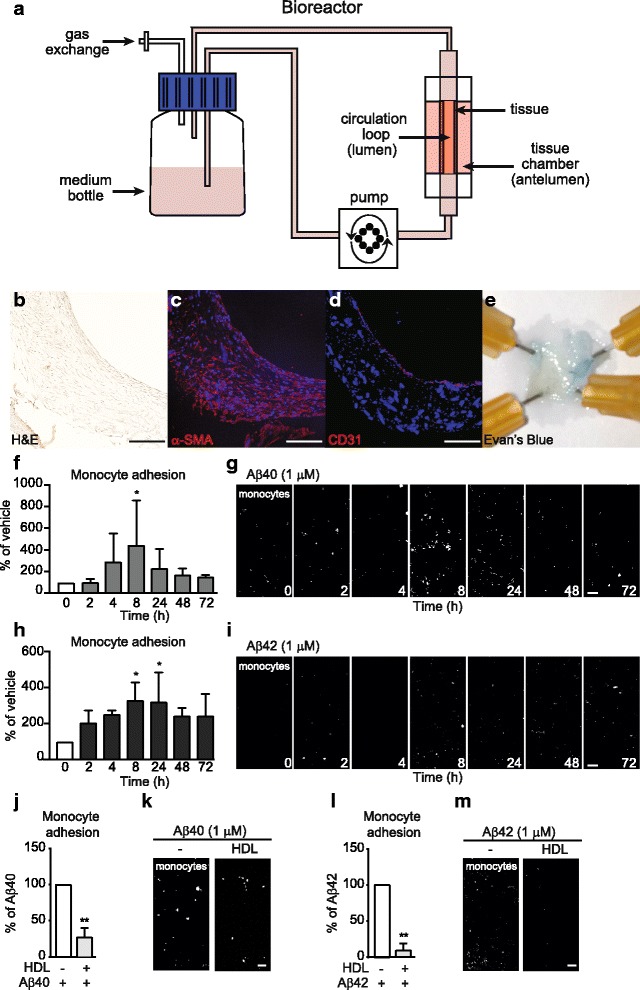
Aβ induces monocyte adhesion in engineered vessels, which is suppressed by HDL. **a** Schematic representation of bioengineered tissue. **b** Histological structure of engineered tissue using hematoxalin-eosin staining to reveal a dense tissue formation composed of cells and extracellular matrix in engineered vessels. **c** α-smooth muscle actin (α-SMA) confirmed the smooth muscle phenotype of cells in the inner layers and **d** CD31 confirmed an endothelial monolayer. Scale bar represents 200 μm. **e** Evans blue staining confirmed a tight endothelium. **f-g** 1 μM of Aβ40 or **h-i** Aβ42 monomers were injected within the tissue chamber (abluminal). Fluorescently labeled human monocytes (THP1, *white*) were circulated in the lumen of the engineered vessels. Tissues were counterstained with DAPI (*blue*) and analyzed using confocal microscopy over time. **j-m** 200 μg/mL of HDL were circulated through the lumen of the grafts for 2 h prior injection of 1 μM of **j-k** Aβ40 or **l-m** Aβ42 monomers within the tissue chamber (abluminal) for 8 h prior to circulating fluorescent THP1 in the lumen. Graphs represent means ± SD of adhered monocytes relative to Aβ treated tissues from at least 3 individual grafts. *p < 0.05, **p < 0.01

Vascular grafts were perfused with complete EGM-2 with 2% FBS. 1 μM Aβ42 or Aβ40 monomers were injected directly into the graft chamber to mimic Aβ originating from the brain (antelumen) side of the vessel. At time points ranging from 2 to 72 h, THP1 cells were fluorescently labeled with Cell-Tracker Red as described above, injected in the graft circulation at a concentration of 1 × 10^6^ cells/mL and maintained under flow conditions for 3 h. For HDL experiments, vascular grafts were perfused with luminal HDL (200 μg/mL) for 2 h before injecting Aβ in the antelumen side for 8 h. Tissues were longitudinally cut open, washed extensively with PBS and fixed with 4% PFA. After 20 min, tissues were washed 3 times with PBS and mounted in Prolong Gold antifade reagent with DAPI. For each independently seeded tissue, adherent monocytes and monocytes undergoing diapedesis were counted in 3 random squares of 1.23 mm^2^ using a z-stack covering the whole tissue thickness with a SP8 confocal microscope (Leica), averaged and expressed as percent of vehicle normalized to 100% for Fig. 1d and f or percent of Aβ normalized to 100% for Fig. 1 h and j, and Fig. 8a and b.

### Static monotypic cell culture

hCMEC/D3 (Fisher; passage 27–35), and HUVEC (passage 4–7, isolated as described [17]) cells were cultured using complete EGM-2 with 2% FBS, ECs were cultured in a humidified incubator at 37 °C at 5% carbon dioxide. For mechanistic experiments, ECs were treated with SR-BI blocking antibody (NB440–113 Novus, 1:500), the SR-BI inhibitor block lipid transport-1 (BLT-1, SigmaAldrich, 10 μM), the eNOS inhibitor L–NG-nitroarginne methyl ester (L-NAME, SigmaAldrich, 1 mM), the receptor associated protein (RAP, Oxford biome, 1 μM), the RAGE blocking antibody (176,902, R&D Systems, 1:50), heparin (10 mU), heparinase (SigmaAldrich, 0.2 mM) or the CD36 blocking antibody (JC63.1, ABCAM, 1:500) for 1 h before HDL priming. For miRNA experiments, cells were transiently transfected using Lipofectamine 2000 (Life Technology) 2 h before Aβ stimulation with the miR-223 mimetic (Life Technology, 4,464,066 (MC12301), 100 nM) or miR-223 inhibitor (Life Technology, 4,464,084 (MH12301), 100 nM) in EBM-2 containing 0.2% BSA.

### Monolayer PBMC adhesion assay

ECs were seeded at 1 × 10^5^ cells/well in 24-well plates and cultured until confluent for 2 to 3 days. On the day of the assay, ECs were primed for 2 h with 100 μg/mL bovine serum albumin as vehicle control or 100 μg/mL HDL before stimulation with 1 ng/mL of TNF-α (Preprotech) or various concentrations of monomeric Aβ40 and Aβ42 (0.001–1 μM, California Peptide), prepared as described below. After 3 h, 5 × 10^5^ Cell-Tracker Red-labeled PBMC per well were added to ECs for 3 additional hours before washing 3 times with PBS to remove non-adherent PBMC. Cells were then fixed with 4% PFA for 15 min before 3 additional PBS washes and 4′,6-diamidino-2-phenylindole (DAPI) counterstaining. For each independent experiment, adherent monocytes were counted in 5 random squares of 7.84 mm^2^ using a fluorescent inverted microscope (Zeiss), averaged and expressed as percent of vehicle normalized to 100%. DAPI counterstaining was used to ensure endothelial cell coverage.

### Aβ oligomerization/fibrilization and electron microscopy confirmation

Recombinant Aβ40 and Aβ42 peptides (California Peptide) were dissolved in ice-cold hexafluoroisopropanol (HFIP). The HFIP was removed by evaporation overnight. To prepare soluble monomers, the peptide film was reconstituted in DMSO to 5 mM, diluted further to 100 μM in DMEM and used immediately. Oligomers were prepared by diluting the 5 mM DMSO peptide solution in phenol red-free F12 medium (Life Technologies) to a final concentration of 100 μM and incubating for 24 h 4 °C. Fibrils were prepared by diluting the 5 mM peptide solution in 0.1 uM of HCl to a final concentration of 100 μM and incubating for 24 h at 37 °C. Aβ monomer, oligomer and fibril preparations were then either used to stimulate hCMEC/D3 monolayers, as given, or were analysed by Transmission Electron Microscopy (TEM) and dot blot. For TEM, 0.5 μL of 100 μM Aβ preparation was diluted in 2 μL filtered distilled water, spotted onto formvar-coated 200-mesh nickel grids (EM Sciences) and allowed to dry. Grids were then negatively stained with 0.5% aqueous uranyl acetate for 30 s, and viewed on a FEI Tecnai G2 Spirit Transmission Electron Microscope. For dot blot, aliquots of Aβ(0.1 μM) were added to PVDF membrane, which were dried and blocked in 3% skimmed milk PBST. After 1 h, blots were incubated with β-amyloid 1–16 antibody (6E10 Biolegend 1:500), amyloid A11 oligomeric antibody (AB9234 Millipore, 1:1000) or amyloid fibrils OC antibody (AB2286 Millipore, 1:1000) in blocking buffer for 16 h, washed extensively in PBST and incubated with anti-mouse or anti-rabbit (1:1000) secondary antibody in blocking buffer. After 1 h, blots were washed as above and developed using enhanced ECL and a ChemiDoc MP imager.

### Measurement of intracellular NO

ECs were seeded at 5 × 10^5^ cells/well in 6-well plates and cultured for 2 days until confluent in complete EBM-2. Sixteen hours before the assay, ECs were serum-depleted in EBM-2 containing 0.2% FBS. On the day of the assay, ECs were incubated with 100 μg/mL of HDL in serum-depleted EBM-2 medium containing 1 μM of 4,5-diaminofluorescein diacetate (DAF2, Caymanchem) at 37 °C. After 6 h, ECs were washed with PBS, trypsinized, and triazolofluorescein fluorescence was measured (excitation wavelength of 485 nm, emission 538 nm), using an Infinite M200Pro plate reader (Tecan). In addition to DAF2 measurement, the phosphorylation of eNOS at Ser1177 was compared to total eNOS by immunoblotting (below) in cell lysates harvested 15 min after HDL treatment.

### Cell surface biotinylation

ECs were seeded at 5 × 10^5^ cells/well in 6-well plates and cultured for 2 days until confluent. On the day of the assay, ECs were treated with HDL, Aβ or TNF-α as above. After stimulation, EC monolayers were washed twice with ice cold PBS (pH 8), cooled on ice for 15 min and biotinylated with 250 μg/mL EZ-link™-sulfo-NHS-biotin (ThermoFisher Scientific) in PBS (pH 8) at 4 °C. After 1 h the reaction was stopped with a 5 min incubation in DMEM with 10% FBS. After two additional PBS washes, ECs were lysed in RIPA buffer. Following quantification of protein concentration using a BCA assay (Thermofisher Scientific), at least 100 μg of protein were incubated with streptavidin-conjugated sepharose beads (Pierce) at 4 °C overnight. Beads were washed 3 times with RIPA buffer and the recovered proteins were resolved on a SDS-PAGE.

### Monolayer aβ association, binding and uptake

ECs were seeded at 3 × 10^5^ cells/well in 12-well plates and cultured until confluent for 2 to 3 days. On the day of the assay, ECs were primed for 2 h with 100 μg/mL HDL before stimulating with 0.1 μM of Aβ40 and Aβ42 monomers at 37 °C for total association, or at 4 °C for cell surface binding. After 3 h, hCMEC/D3 were washed 3 times with PBC and lysed in RIPA buffer containing 10 mM Tris pH 7.4, 150 mM NaCl, 1.0% NP-40, 1.0````% sodium deoxycholate, 0.1% SDS and cOmplete protease inhibitor with EDTA (Roche). Aβ40 (KHB3442, Life Tech) and Aβ42 (KHB3482, Life Tech) were quantified using commercial ELISAs and normalized to total protein concentration. For Aβ uptake, hCMEC/D3 were seeded at 1 × 10^5^ cells/well in 24-well plates and cultured to confluence for 2 to 3 days. On the day of the assay endothelial, ECs were primed for 2 h with 1 mg/mL of HDL before stimulating with 1 μM monomeric FITC-Aβ40 and FITC-Aβ42 (Bachem) prepared as described above. After 3 h at 37 °C, hCMEC/D3 were washed 3 times with PBS and fixed in 4% PFA for 20 min. After one Tris-HCl and two PBS washes, hCMEC/D3 were mounted in Prolong antifade reagent.

### Molecular biology

For mRNA, cells were lysed in Trizol (Invitrogen) and RNA was extracted and treated with DNase I (Invitrogen) according to the manufacturer’s protocol. cDNA was generated using oligo-dT primers and Taqman reverse transcription reagents (Applied Biosystems). Real-time quantitative PCR was done using FastStart Universal SYBR Green Master reagent (Roche) on a Light Cycler 96 system (Roche) to quantify gene expression relative to vehicle using specific primer against ICAM-1 (fwd: ATGGCAACGACTCCTTCTCG; rev: CGCCGGAAAGCTGTAGATGG) and VCAM-1 (fwd: TGTTTGCAGCTTCTCAAGCTTTT; rev: GATGTGGTCCCCTCATTCGT) and normalized to GAPDH (fwd: CCTGCACCACCAACTGCTTA; rev: CATGAGTCCTTCCACGATACCA). MiRNA was isolated using an miRNeasy mini kit (Qiagen) following the manufacturer’s instructions, respectively. Reverse transcription was performed using 2 μg of total RNA with specific miRNA primers (Life Technology). MiR223 and U6 were quantified using specific TaqMan probes (Life Technology).

### Immunoblot

ECs were lysed in RIPA buffer containing either cOmplete protease inhibitor or Phosphostop (Roche) and quantified using a BCA assay. Equal amounts of total protein were separated by SDS-PAGE followed by electrophoretic transfer to polyvinylidene fluoride (PVDF) membranes (Millipore). After blocking membranes for 1 h with 5% skim milk powder in PBST, or 5% BSA in TBST for phosphoproteins, ICAM-1, (EP1442Y Abcam, 1:1000), VCAM-1 (EPR5047 Novus, 1:1000), peNOS (ser1177 Cellsignalling, 1:1000), eNOS (M221 ABCAM, 1:1000), AnxA1 (D5V2T, Cellsignalling, 1:1000), p-Akt (Ser473 D9E, Cellsignalling, 1:2000), Akt (9272, Cellsignalling, 1:1000), p-NF-κB p65 (Ser536 93H1, Cellsignalling, 1:1000), NF-κB p65 (D14E12, Cellsignalling, 1:1000), p-SAPK/JNK (Thr183/Tyr185 G9, Cellsignalling, 1:1000), JNK (2C6, Cellsignalling, 1:1000), p-p42/44 MAPK (Erk1/2) (Thr202/Tyr204 D13.14.4E, Cellsignalling, 1:1000), p42/44 MAPK (Erk1/2) (137F5, Cellsignalling, 1:1000), p-Stat3 (Tyr705, Cellsignalling 1:1000), Stat3 (124H6, Cellsignalling, 1:1000) and GAPDH (MAB374 1:10,000, Millipore) were immunodetected by incubating for 16 h in primary antibody in blocking buffers. Membranes were washed extensively with PBST or TBST, and incubated with anti-mouse or anti-rabbit (1:1000–10′000, Jackson ImmunoResearch) secondary antibody in blocking buffer. After 1 h, membranes were washed as above and developed using enhanced chemiluminescence (ECL, Amersham) and a ChemiDoc MP imager (Biorad). Densitometric images were captured with ImageJ and band intensity normalized to GAPDH as a loading control.

### Beta-sheet formation assay

Cell-free Thioflavin-T fibrillization assays were performed on an Infinite M2000 Pro plate reader (Tecan) as described (Truran 2015). Briefly, 10 μM monomeric Aβ40 or Aβ42 were incubated in a buffer consisting of 20 mM of Thioflavin-T in 150 mM NaCl and 5 μM of HEPES at pH 7.4, with and without 1 mg/mL of HDL, at 37 °C with 20 s of orbital shaking (3 mm amplitude) every 5 min in a black 96-well plate. Formation of fibrillar β-amyloid pleated sheets over time was monitored by excitation at 440 nm and measuring emission intensity at 490 nm every 5 min up to 12 h in total.

### Human brain protein extraction and ELISA

Frozen brain tissues (cortex Brodmann area 9 and cerebellum) were provided by the Harvard Brain Tissue Resource Center under an approved UBC protocol (C04–0595) and extracted with 8 volumes of ice-cold carbonate buffer (100 mM Na_2_CO_3_, 50 mM NaCl, pH 11.5) containing cOmplete protease inhibitor (Roche Applied Science) by manual homogenization with a tissue probe. After incubating on ice for 10 min, lysates were clarified by centrifugation at 16,600 rcf for 45 min at 4 °C. The supernatant was removed and neutralized by adding 1.5-volumes of 1 M Tris-HCl pH 6.8 to give a final pH of approximately 7.4. Brain tissues from all samples were extracted in an identical manner, and fractions were aliquoted and immediately frozen at −80 °C until analysis. Protein concentrations were determined using the Lowry Protein Assay (Biorad). ICAM-1 (ab174445, ABCAM), VCAM-1 (ab187393, ABCAM), Aβ40 (KHB3442, Life Tech) and Aβ42 (KHB3482, Life Tech) in carbonate extracts were quantified using commercial ELISAs. ELISA data were normalized to total protein concentration of the extract. For immunofluorescent staining, brains were sectioned at 20 μm, rehydrated in PBS and blocked in 4% paraformaldehyde (PFA). After 20 min, sections were washed once with Tris-HCl (0.5 mM, pH 8), twice with PBS, and blocked in blocking buffer (5% goat serum and 1% BSA). After 60 min, section were incubated at 4 °C with antibodies against CD31 (WM59 Biolegend, 1:50), ICAM-1 (EP1442Y ABCAM 1:200), and VCAM-1 (EPR5047 ABCAM 1:200) in blocking buffer overnight. Sections were washed 3 times in PBS and incubated at RT with Alexa-488 anti-mouse and Alexa-594 anti-rabbit fluorescently labeled secondary antibodies (LifeTechnologies, 1:600). After 45 min and 3 additional PBS washes, sections were mounted in Prolong Gold antifade reagent with DAPI (LifeTechnologies) and imaged on an inverted fluorescent microscope (Zeiss)

### Statistical analysis

All statistical analyses were performed using SPSS and *p*-values <0.05 were considered significant. Data were obtained from at least 3 independent experiments and are presented as mean ± SD if not indicated otherwise. Data were first log transformed and analyzed by two-way ANOVA with a blocking factor (experiment) with direct comparison when comparing two treatments or Dunnett’s or Bonferonni multi-comparison tests. After statistical calculations, vehicle data were normalized to 100% and represented as a dashed line in the graph, and tested conditions were expressed and graphed as percentage of vehicle if not otherwise stated.

## Results

### HDL suppresses aβ-mediated monocyte adhesion to ECs in three-dimensional dynamic engineered human vessels

To mimic the complexity of native cell-cell and/or cell-matrix interactions observed in native human vessels, we used innovative tissue engineering technology to generate in vitro 3D human vessels composed of primary human umbilical vein ECs (HUVEC) and primary human smooth muscle cells (SMC) to maximize translational relevance of our studies relative to in vitro studies that solely use traditional static cell culture models. Our system uses a scaffold-directed dynamic, semi-pulsatile flow bioreactor system, on which primary human cells are sequentially seeded into 2 mm diameter biodegradable polyglycolic-acid (PGA)/polycaprolactone (PCL) composite matrices. After a short static incubation, vascular constructs are exposed to dynamic flow in a bioreactor, where nutrient medium is directed through the lumen of the bioreactor circulation loop to mimic native blood flow. We previously demonstrated that these scaffold-directed engineered vessels are useful for studies of endothelial activation in 3D culture [17]. A schematic of the bioreactor system is presented in Fig. 1a. Under our flow bioreactor conditions, haematoxylin and eosin staining confirmed a dense tissue formation composed of cells with extracellular matrix on the luminal side of the scaffold (Fig. 1b-d). Immunofluorescent staining confirmed a SMC phenotype of cells in the inner layers and an EC monolayer on the luminal side (Fig. 1c,d). Integrity of the endothelial barrier was functionally assessed by injecting Evans blue dye into the bioreactor circulation loop, which was excluded after EC seeding, demonstrating a functionally tight endothelial barrier (Fig. 1e).

Having established a 3D human vascular model, we injected 1 μM Aβ40 or Aβ42 directly into the graft chamber to mimic Aβ coming from the brain anteluminal side, followed by injection of human THP-1 monocytes into the lumen chamber to mimic circulating monocytes. Both Aβ40 and Aβ42 led to THP1 adhesion to endothelium in the engineered vessels, with the most robust response appearing 8 h after Aβ injection (Fig. 1f-h). We then tested the ability of HDL, isolated from healthy human donors using KBr density gradient ultracentrifugation, to suppress Aβ-induced monocyte adhesion to ECs in engineered vessels by circulating either media alone or 200 μg/mL of HDL for 2 h prior to injecting 1 μM Aβ into the graft chamber. In this engineered vascular model, HDL robustly suppressed monocyte adhesion to endothelium by both Aβ40 (5 fold, *p* = 0.0286) and Aβ42 (10 fold, *p* = 0.0016) **(**Fig. 1i-l).

### HDL attenuates aβ-induced peripheral blood mononuclear cell (PBMC) adhesion in monotypic ECs

**Fig. 2:**
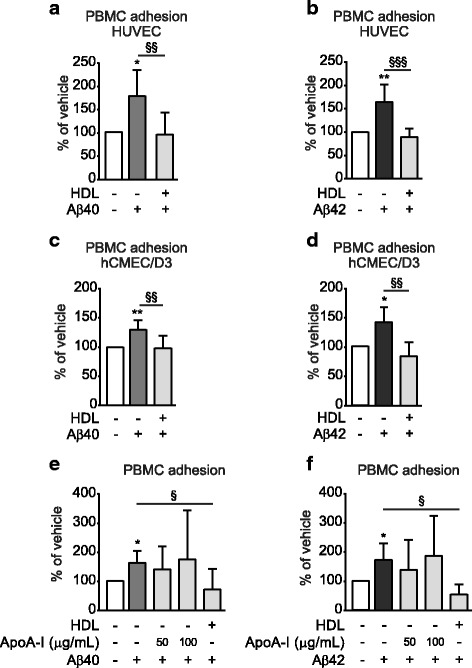
Aβ40 and Aβ42 induce PBMC adhesion to ECs, which is suppressed by HDL. HUVEC (**a-b**) or hCMEC/D3 **(c-d**) were primed with 100 μg/mL of HDL and stimulated with 0.1 μM **a, c** Aβ40 (*light grey*) or **b, d**Aβ42 (*dark grey*) monomers for 3 h. Fluorescently labelled PBMC were allowed to adhere to stimulated cells for 3 h followed by washing, fixation, imaging, and counting. hCMEC/D3 were primed with either 100 μg/mL of HDL or 50 μg/mL or 100 μg/mL lipid-free human apoA-I for 2 h followed by stimulation with 0.1 μM **e** Aβ40 (*light grey*) or **f** Aβ42 (*dark grey*) monomers for 3 h. Cells were washed, imaged and counted as above. Graphs represent mean ± SD of adhered PBMC relative to vehicle control from at least 3 independent trials where * *p* < 0.05, **p < 0.01, ***p < 0.001 versus vehicle, § *p* < 0.05, §§ *p* < 0.01 versus Aβ

To define the mechanisms by which HDL attenuates Aβ-induced monocyte binding to ECs, we used static monolayer EC cultures and peripheral blood mononuclear cells (PBMC) isolated by continuous density gradient ultracentrifugation from healthy human donors. We first confirmed that Aβ induces PBMC adhesion in monotypic HUVEC cultures and that HDL attenuates this activity (Fig. 2a,b). As Aβ originates within the brain and the primary pathways by which Aβ is cleared from the brain involve cerebral vessels [7], we also showed that the human brain microvascular endothelial cell line hCMEC/D3, a commonly used cell line for in vitro studies of the blood brain barrier (BBB) [20], also exhibit increased PBMC adhesion upon Aβ treatment and that this too is attenuated by HDL (Fig. 2c,d). As HUVEC and hCMEC/D3 give nearly identical results, we focused on hCMEC/D3 cells for all subsequent mechanistic experiments.

Our first question was to determine if lipid-free apoA-I was functionally equivalent to mature HDL in its ability to suppress Aβ-mediated PBMC binding to hCMEC/D3. Our results clearly showed that pre-incubation of hCMEC/D3 with lipid-free apoA-I (50 and 100 μg/mL) did not alter Aβ-induced PBMC adhesion (Fig. 2e,f), demonstrating that mature HDL particles are required to attenuate Aβ-induced PBMC adhesion to brain-derived ECs.

To establish the experimental conditions to investigate how HDL suppresses Aβ-mediated PBMC adhesion to hCMEC/D3, we performed PMBC adhesion assays in hCMEC/D3 after a 3 h exposure to increasing concentrations human Aβ40 and Aβ42 monomers. Compared to baseline, Aβ40 concentrations of 0.01, 0.1 and 1.0 μM led to significant 175%, 169% and 179% increases in PMBC adhesion (*p* = 0.033, 0.002 and 0.019 respectively, Additional file 1a), The same concentrations of Aβ42 also led to significant 184%, 161% and 223% increases in PMBC adhesion (*p* = 0.019, 0.0406 and 0.0421, respectively) relative to baseline (Additional file 1b). Having demonstrated that 0.1 μM of monomeric Aβ40 or Aβ42 is sufficient to induce PMBC adhesion in hCMEC/D3, we then tested whether pre-treating hCMEC/D3 for 2 h with 0 to 400 μg/mL HDL could attenuate Aβ-induced PBMC adhesion. Significant suppression of PBMC adhesion was observed at concentrations of HDL from 100 μg/mL and above**.** At 100 μg/mL HDL there was an 80% and 60% reduction of adhered PBMCs in cells induced by 0.1 μM Aβ40 (*p* = 0.017) or 0.1 μM Aβ42 (*p* = 0.018) respectively (Additional file 1c,d).

### HDL reduces the fibrillization rate of aβ42 and aβ40 in cell-free conditions

**Fig. 3:**
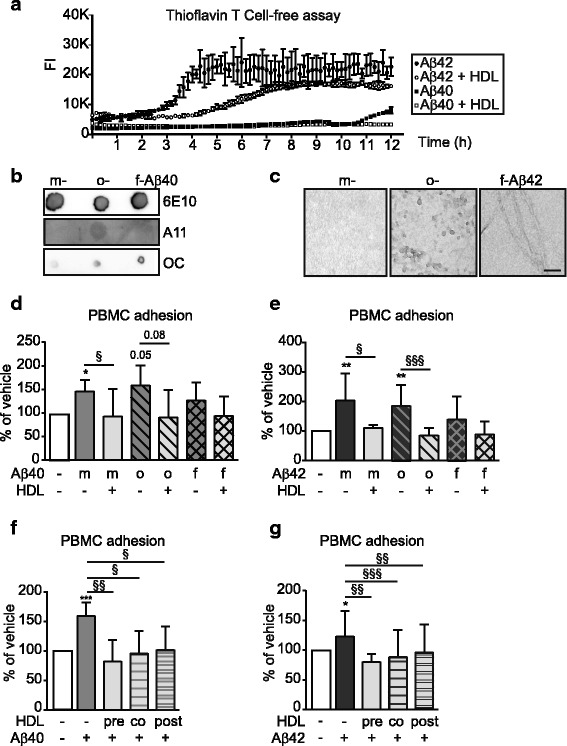
HDL delays beta-sheet formation and attenuates Aβ-induced PBMC adherence independent of Aβ structure. **a** Representative graph ± SD of technical triplicates of 3 individual experiments where 10 μM Aβ40 or Aβ42 with or without 10 mg/mLl HDL were incubated with 20 μM of Thio-T in 150 mM NaCl and 5 μM of HEPES (pH 7.4) for 12 h at 37 °C. Formation of β-amyloid pleated sheets was monitored every 5 min at excitation 440 nm and emission 490 nm. **b-c** Aβ structures were confirmed using dot blot with antibodies against oligomers (A11) or fibrils (OC) and electron microscopy (EM). **d-e** hCMEC/D3 were stimulated for 3 h with 0.1 μM monomeric (m-Aβ, *solid bar*), oligomeric (o-Aβ, *striped bar*) or fibrillar-aggregated (f-A Aβ, *cross-hatched bar*) **d** Aβ40 or **e** Aβ42 in the presence or absence of HDL. **f-g** hCMEC/D3 were pre-incubated for 2 h with 100 μg/mL HDL prior to Aβ addition (pre, *solid bar*), co-incubated with 100 μg/mL HDL and Aβ (co, *striped bar*), or post-incubated by adding 100 μg/mL HDL 1 h following Aβ stimulation (post, *double striped bar*). Graphs represent means ± SD of adhered PBMC relative to vehicle treated cells from at least 5 independent trials where. *p < 0.05, **p < 0.01, ***p < 0.001 * *p* < 0.05, **p < 0.01, ***p < 0.001 versus vehicle, § *p* < 0.05, §§ *p* < 0.01, §§§*p* < 0.001 versus Aβ

As Aβ has been reported to bind HDL-like particles in both cerebrospinal fluid (CSF) and blood [21], and apoA-I has been reported to affect Aβ aggregation [13, 22], we reasoned that one mechanism by which HDL may attenuate PBMC adhesion to hCMEC/D3 could be by affecting Aβ structure to prevent the formation of toxic higher order species [23]. A cell-free Thioflavin-T reporter assay was used to determine whether HDL affects the fibrillization kinetics of Aβ40 or Aβ42 over a 12 h period, using 10 μM Aβ and 10 mg/mL HDL (ratio: 1:1), which is the same Aβ:HDL ratio used for our cellular PBMC adhesion assays and allows detection of fluorescence signal using a plate reader (Fig. 2c,d). Under these conditions, in the absence of HDL, the onset of Aβ fibrillization requires at least 3 h for Aβ42 and at least 10 h for Aβ40. Notably, in the presence of HDL, we observed delayed onset of fibrillization for both Aβ42 and Aβ40, and a slower rate of Aβ42 fibrillization (Fig. 3a). As HDL can suppress PBMC adhesion to hCMEC/D3 induced by both Aβ40 and Aβ42 within 3 h of Aβ addition, we conclude that suppression of Aβ fibrillization cannot be the only mechanism by which HDL functions to protect ECs from the detrimental effects of Aβ.

### Aβ structure does not affect HDL’s ability to reduce PBMC adhesion to hCMEC/D3

Several experiments were then performed to further test whether Aβ structure affects HDL’s anti-inflammatory activity on hCMEC/D3. We varied the structural species of the Aβ preparation added to hCMEC/D3, reasoning that if HDL acts primarily through maintaining Aβ in a soluble monomeric state, the protective effect of HDL should be diminished if ECs are stimulated with preformed Aβ oligomers or fibrils. Soluble monomeric and oligomeric, and insoluble fibrillar Aβ were prepared as described [24] and structures were confirmed using a dot blot assay as well as electron microscopy (Fig. 3b,c). Interestingly, monomeric and oligomeric Aβ40 and Aβ42 preparations induced comparably robust PBMC adherence, whereas the insoluble fibrillary form failed to significantly increase PBMC adhesion. Importantly, the ability of HDL to suppress PMBC adhesion was unaffected by input of either Aβ monomers or oligomers (Fig. 3d-e). These results suggest that delayed Aβ fibrillization may not be the major pathway by which HDL suppresses PBMC adhesion to ECs under our experimental conditions as HDL maintained its protective effect when ECs are treated with pre-formed oligomers. To confirm this, we also varied the timing of HDL and Aβ addition to hCMEC/D3 such that, in addition to a 2 h HDL pre-incubation as above, HDL was added at the same time as Aβ (co-incubation) or 1 h after Aβ addition (post-incubation). We reasoned that if HDL acts primarily through maintaining Aβ solubility, pre-incubation and co-incubation designs should give equivalent and maximal suppression of Aβ-induced PBMC adhesion as, in both scenarios, Aβ is always in the presence of HDL and aggregation could be delayed. In contrast, as the post-incubation scenario would allow Aβ aggregation to begin prior to addition of HDL, the protective effect of HDL should be reduced if added after the Aβ stimulus if Aβ structure is the key driver of PBMC adhesion to ECs. We observed that PMBC adhesion was significantly and similarly reduced regardless of when HDL was added to hCMEC/D3 relative to Aβ40 or Aβ42 providing additional support that HDL’s ability to attenuate Aβ-mediated PBMC adhesion to hCMEC/D3 is independent of whether Aβ input is monomeric or oligomeric (Fig. 3f,g).

These two lines of evidence suggest that, although HDL can affect Aβ structure in cell-free conditions, these effects are neither rapid nor robust enough to fully explain how HDL suppresses Aβ mediated PBMC adhesion to hCMEC/D3. We next evaluated signalling pathways implicated in HDL’s anti-inflammatory effects on ECs.

### The ability of HDL to suppresses aβ-induced PBMC adhesion to hCMEC/D3 is independent of nitric oxide (NO) production and Annexin A1 (Anx1)

**Fig. 4:**
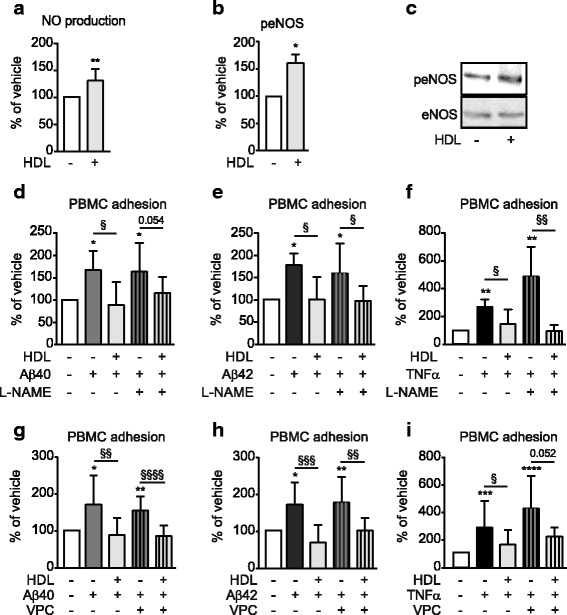
HDL suppression of Aβ-induced inflammation is independent of eNOS and S1P. **a** Intracellular NO production was measured by treating hCMEC/D3 with 100 μg/mL HDL in the presence of 1 μM DAF-2 for 6 h. Fluorescence was measured at 485 nm. **b** Phosphorylation of eNOS was measured by treating hCMEC/D3 with 100 μg/mL HDL for 15 min before immunoblotting for phosphorylated eNOS (p-eNOS) or total eNOS. Representative immunoblots are shown in (**c**). hCMEC/D3 were pretreated for 1 h with the eNOS inhibitor L-NAME (**d-f**) or the S1P1 and S1P3 inhibitor VPC23019 (**g-i**) followed by 100 μg/mL HDL for 2 h. Cells were then stimulated with 0.1 μM Aβ40 (**d,g)** Aβ42 monomers, **(e,h)** or 1 ng/mL of TNF-α (**f,i**) for 3 h before testing PBMC adherence. Graphs represent means ± SD of adhered PBMC relative to vehicle treated cells for at least 5 independent trials. *p < 0.05, **p < 0.01, ***p < 0.001 * *p* < 0.05, **p < 0.01, ***p < 0.001 versus vehicle, § *p* < 0.05, §§ *p* < 0.01, §§§*p* < 0.001 versus Aβ or TNF-α

Stimulation of NO synthesis by HDL is reported to reduce arterial EC activation [25]. We therefore tested whether HDL induces NO production in hCMEC/D3 using the cell permeable indicator 4,5-Diaminofluorescein diacetate (DAF2), which reacts with NO to produce highly fluorescent trizaolo-fluorescein. Compared to baseline conditions, HDL-treated hCMEC/D3 showed a significant 130% increase (*p* = 0.01) in NO production (Fig. 4a), as well as significantly elevated phosphorylated endothelial NO synthase (eNOS) on serine 1177 (161%, *p* = 0.047) (Fig. 4b,c). These results confirm that HDL can induce NO production in hCMEC/D3, as observed in ECs derived from other origins.

To test whether NO generation is required for HDL to suppress Aβ-mediated PBMC adhesion to hCMEC/D3, we treated cells with the eNOS inhibitor L-NAME, followed by the addition of HDL, and finally stimulation with either Aβ40, Aβ42 or TNF-α. We observed that blocking eNOS with L-NAME had no effect on the ability of HDL to suppress either Aβ- or TNF-α-mediated PBMC adhesion to hCMEC/D3 (Fig. 4d-f). Additional experiments confirmed the potency of L-NAME to block eNOS in HDL-stimulated HUVEC (Additional file 2a), yet this also had no effect on the ability of HDL to suppress either Aβ- or TNF-α- mediated PBMC binding in HUVEC (Additional file 2b–d). We further ruled out a role for the NO pathway in hCMEC/D3 by measuring the effect of HDL in the presence of VPC23019, an antagonist of sphingosine-1 phosphate receptor 1 (S1P1) and S1P3, receptors that are required for eNOS activity [26], and again found no effect on the ability of HDL to suppress either Aβ- or TNF-α-mediated PBMC adhesion to hCMEC/D3 (Fig. 4g-i) or HUVEC (Additional file 2e–h). Taken together, these results provide strong support that the eNOS pathway is not involved in the ability of HDL to suppress PBMC adhesion to Aβ- or TNF-α-activated hCMEC/D3 and HUVEC.

In contrast to a recent report suggesting that HDL up-regulates Anx1, which affects HDL’s ability to suppress ECs activation [27], we observed no significant change in Anx1 protein levels in hCMEC/D3 after either HDL or Aβ treatment (Additional file 2i).

### HDL-mediated suppression of aβ-induced PBMC adhesion to hCMEC/D3 is independent of miR-233

In addition to numerous proteins and lipid species, HDL particles carry microRNAs (miRNAs) that can correlate with vascular disease risk [28]. One of the most abundant miRNAs in HDL is miR-233, which has recently been shown to affect gene expression in human coronary artery ECs including suppressing ICAM-1 expression [29]. Three lines of evidence suggest that HDL-mediated suppression of Aβ-mediated PBMC binding to hCMEC/D3 is independent of miR-233. First, direct transfection of a miR-223 mimetic did not prevent Aβ40 or TNF-α-mediated PBMC adhesion to hCMEC/D3, although a statistically significant reduction with Aβ42 was observed **(**Additional file 3a–c). Second, priming hCMEC/D3 with HDL in the presence of a specific miR-223 inhibitor did not diminish the ability of HDL to suppress PMBC adhesion by Aβ40 , Aβ42 or TNF-α (Additional file 3d–f). Third, in the 5 h period where hCMEC/D3 were exposed to HDL (2 h pre-treatment then 3 h with Aβ), no significant transfer of miR-223 was detected (see Additional file 3g), consistent with a previous report that after 6 h, miR-223 was not transferred from HDL to several types of ECs including HUVEC [30]. These results suggest that, although the miR-233 mimetic can suppress Aβ42-mediated PBMC binding to hCMEC/D3 when added directly, miR-233 is unlikely to underlie the ability of HDL to suppress PBMC adhesion to hCMEC/D3 triggered by Aβ40, Aβ42 or TNF-α.

### HDL-mediated suppression of aβ-induced PBMC adhesion to hCMEC/D3 is independent of ICAM-1 and VCAM-1.

ICAM-1 and VCAM-1 are canonical markers of EC activation that mediate PBMC adhesion by classical inflammatory stimuli [31]. Several studies have reported elevated microvascular inflammatory markers in the AD brain [32, 33]. Using ELISA, we confirmed that protein levels of intercellular adhesion molecule 1 (ICAM-1) but not vascular cell adhesion molecule 1 (VCAM-1) are increased in the cortex of AD patients compared to either cerebellum from the same individual or the cortex from non-cognitively impaired (NCI) control subjects (Additional file 4). Increased ICAM-1 levels were further confirmed using immunofluorescence revealing both vascular and glial ICAM-1 reactivity whereas VCAM-1 remained unchanged (Additional file 5a–b). As expected, soluble Aβ40 and Aβ42 levels were elevated in the cortex of AD patients compared to their corresponding cerebellum or to the cortex of NCI subjects (Additional file 4). Because ICAM-1 is a classical marker of EC activation and HDL can attenuate PBMC adherence by reducing ICAM-1 levels in aortic derived ECs [34], we also evaluated the roles of ICAM-1 and VCAM-1 in hCMEC/D3 activation. Using Western blotting, we observed that pretreatment of monotypic hCMEC/D3 cultures with HDL significantly suppressed TNF-α-mediated induction of ICAM-1 (from 1343% to 920%, *p* = 0.041) and VCAM-1 (from 2838% to 1928% *p* = 0.012), relative to untreated cells (Additional file 6a–c), results that are consistent with HDL’s anti-inflammatory effects on peripherally derived ECs. By contrast, Aβ treatment did not affect either mRNA levels or total protein levels of VCAM-1 and ICAM-1 in hCMEC/D3 (Additional file 6d–m). Although total ICAM-1 levels showed no significant change in response to either Aβ40 or Aβ42 treatment, biotinylation assays revealed significantly increased cell surface ICAM-1 levels, but not VCAM-1 levels, in response to 0.1 μM of Aβ40 (*p* = 0.009) and a strong trend in response to Aβ42 (*p* = 0.06) (Additional file 6 h–j). Unexpectedly, however, we observed that cell surface ICAM-1 levels were not reduced by a 2 h exposure to HDL (Additional file 6 k–m), showing that cell surface ICAM-1 levels do not correlate with PMBC adhesion in Aβ-activated hCMEC/D3. We next analysed the effects of Aβ on the phosphorylation of multifunctional serine/threonine protein kinases that are involved in EC activation, survival, apoptosis, proliferation and migration. TNF-α activated NFκB p-P65 (*p* = 0.002), SAPK/JNK (*p* = 0.005), MAPK/ERK (*p* = 0.01), but not Akt or STAT2 pathways, whereas Aβ did not alter any of these pathways in hCMEC/D3 (Additional file 7). Taken together, these results suggest that Aβ does not activate any of classical inflammatory pathways known to increase PBMC adhesion through ICAM-1 or VCAM-1.

### Suppressing aβ uptake into hCMEC/D3 blocks PBMC adherence

**Fig. 5:**
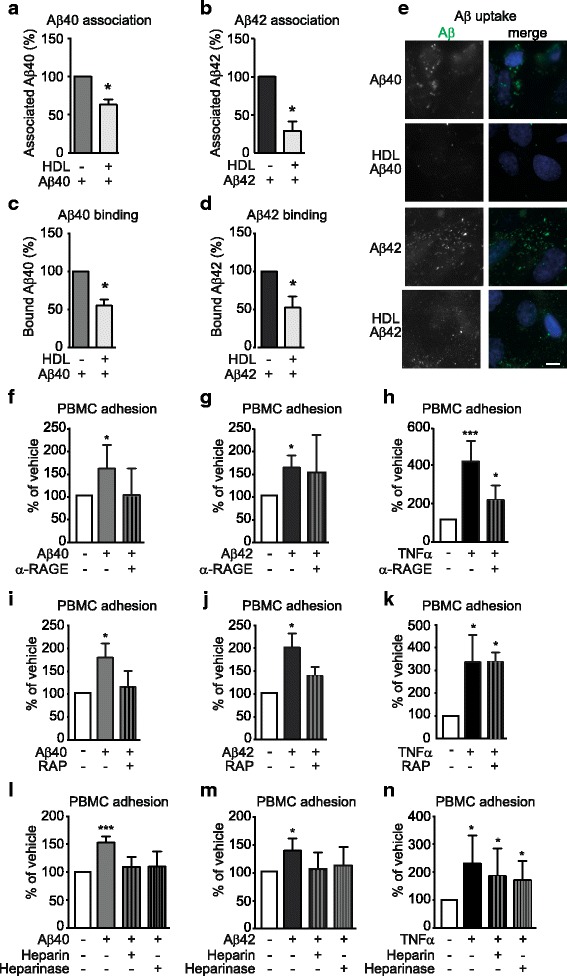
HDL reduces Aβ association, binding and uptake to hCMEC/D3 whereas blocking Aβ binding or uptake reduces PBMC adhesion to hCMEC/D3. **a-d** hCMEC/D3 were pre-treated with 100 μg/mL of HDL and 0.1 μM **a,c** Aβ40 or **b,d** Aβ42 monomers as described in Fig. 2 at either 37 °C (association **a,b**) or at 4 °C (binding **c,d**). Cells were lysed in RIPA buffer and Aβ were measured using commercial ELISA. **e** hCMEC/D3 were pre-treated with HDL (1 mg/mL) for 2 h before stimulating with 1 mM of fluorescently labelled Aβ40 or Aβ42 monomers. Scale bar represents 10 μm. **f-n** hCMEC/D3 were pre-treated with (**f-h**) RAGE blocking antibody, **i-k** RAP or **l-n** heparin or heparinase III 60 min before stimulation with Aβ40 or Aβ42 monomers or TNF-α for 3 h. PBMC adhesion assays were conducted as described in Fig. 2. Graphs represent means ± SD relative to vehicle treated cells for at least 3 independent trials. *p < 0.05, **p < 0.01 versus vehicle

As our results show that Aβ activates hCMEC/D3 through a mechanism that is independent of canonical EC activation intracellular signalling pathways, we hypothesised that binding or uptake of Aβ to ECs might influence PBMC adhesion. To test this hypothesis, we first used temperature modulation experiments to investigate how HDL affects the interactions of Aβ with hCMEC/D3 and observed that HDL pre-treatment significantly reduced total association measured at 37 °C (Fig. 5a,b), cell surface binding measured at 4 °C measured by ELISA (Fig. 5c,d) and intracellular uptake of fluorescently labeled Aβ40 and Aβ42 (Fig. 5e). Second, we tested whether blocking RAGE and LRP1, known receptors that modulate Aβ binding and uptake in ECs [35], might reduce PBMC adhesion. Blocking RAGE with a specific antibody abrogated PBMC adhesion to both Aβ40 and Aβ42 but not TNF-α stimulated hCMEC/D3(Fig. 5f-h). Similarly, blocking LRP1 with receptor associated protein (RAP) abolished Aβ40 and Aβ42 but not TNF-α effects (Fig. 5i-k). Finally, as heparin sulphate proteoglycans (HSPG) are also involved in binding and uptake of Aβ in neuron [36], we then saturated or removed HSPG by treating hCMEC/D3 with heparin or heparinase III, respectively. Both treatments abolished the ability of Aβ40 and Aβ42 to induce PBMC binding whereas TNF-α induced-PBMC adhesion remained significant (Fig. 5l-n). Together, these observations show that, unlike TNF-α, Aβ induces PBMC adhesion to ECs through a pathway that requires interactions with or internalization through Aβ receptors on the EC surface. We also observed that HDL does not decrease the expression of RAGE or LRP1 (Additional file 8), suggesting that down-regulation of these receptors cannot explain the protective effect of HDL on Aβ-induced PBMC binding to hCMEC/D3.

### Scavenger receptor-BI (SR-BI) is necessary for HDL to suppress aβ-induced PBMC adhesion to ECs

**Fig. 6:**
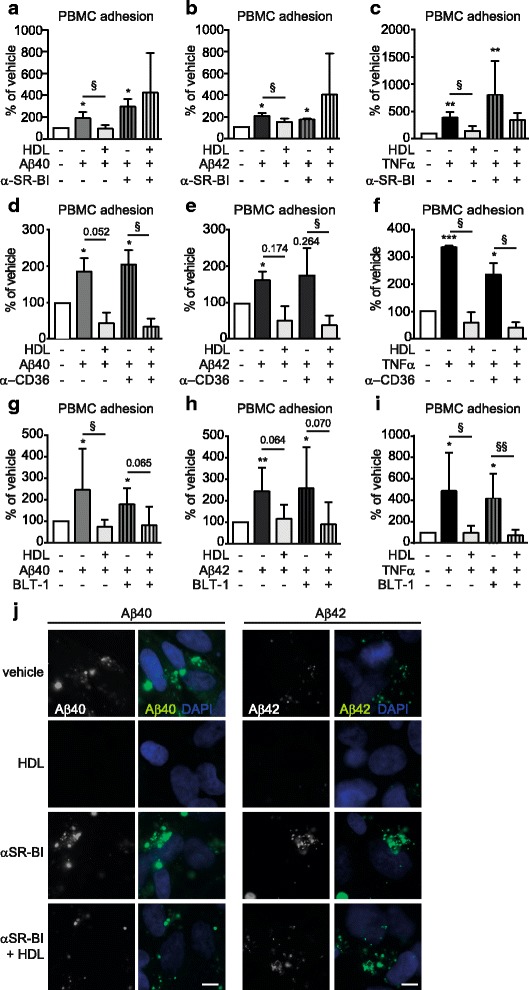
HDL suppression of Aβ-induced inflammation requires SR-BI. hCMEC/D3 were pre-treated for 1 h with **a-c** SR-BI or **d-f** CD36 blocking antibodies or **g-i** BLT1 followed by 100 μg/mL HDL for 2 h. Cells were then stimulated with 0.1 μM monomeric **a,d,g** Aβ40 or **b,e,h** Aβ42 or (**c,f,i**) 1 ng/mL of TNF-α for 3 h before evaluating PBMC adherence. **j** hCMEC/D3 were pre-treated for 1 h with SR-BI blocking antibody followed by HDL (1 mg/mL) for 2 h before stimulating with 1 mM of fluorescently labelled Aβ40 or Aβ42 monomers. Scale bar represents 10 μm. Graphs represent means ± SD of adhered PBMC relative to vehicle treated cells from at least 4 independent trials. *p < 0.05, **p < 0.01, ***p < 0.001 * *p* < 0.05, **p < 0.01, ***p < 0.001 versus vehicle, § *p* < 0.05 versus Aβ or TNF-α.

Scavenger receptor BI (SR-BI) is the principal HDL receptor on ECs and activates several HDL signalling pathways in addition to mediating selective cholesterol uptake upon HDL docking [37]. We observed that inhibiting SR-BI binding with a specific blocking antibody abolished the ability of HDL to suppress both Aβ- and TNF-α-mediated PBMC adhesion to hCMEC/D3 (Fig. 6a-c). Selectivity to SR-BI was confirmed by demonstrating that blocking scavenger receptor CD36 with a specific antibody had no effect on the ability of HDL to suppress either Aβ- or TNF-α-mediated PBMC adhesion to hCMEC/D3 (Fig. 6d-f). Intriguingly, inhibiting SR-BI-mediated selective cholesterol uptake with block lipid transfer 1 (BLT1) did not affect the ability of HDL to suppress either Aβ- or TNF-α-mediated PBMC adhesion to hCMEC/D3 (Fig. 6g-i). We further observed that inhibiting SR-BI with a specific blocking antibody abolished the ability of HDL to suppress Aβ uptake in hCMEC/D3 (Fig. 6j). These results demonstrate that HDL requires SR-BI-mediated signalling to inhibit Aβ uptake and subsequently suppress PBMC adhesion to hCMEC/D3 in response to Aβ- or TNF-α in a manner independent of lipid uptake.

**Fig. 7:**
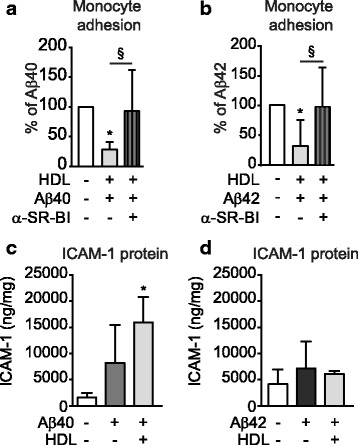
HDL suppresses Aβ-induced monocyte adhesion in engineered vessels via SR-BI. SR-BI blocking antibody was circulated through the lumen of engineered vessels prepared using HUVEC for 1 h prior treatment with 200 μg/mL of HDL for 2 h followed by injection of 1 μM of monomeric **a** Aβ40 or **b** Aβ42 within the tissue chamber (abluminal) for 8 h prior to injecting circulating fluorescent THP1 into the lumen. **c-d** ICAM-1 protein was measured in tissue lysates prepared in RIPA buffer by commercial ELISA. Graphs represent means ± SD of adhered monocytes relative to Aβ treated tissues from at least 4 individual grafts. * and # *p* < 0.05

Having used monotypic static cell cultures to demonstrate that HDL suppresses Aβ-induced PBMC adhesion to endothelial cells through a mechanism that requires SR-BI and is independent of ICAM-1 expression, we confirmed that this mechanism also explains the results in our 3D bioengineered vessels. We observed that inhibiting SR-BI with a specific antibody circulated through the vessel lumen abolished the ability of HDL to suppress Aβ-mediated monocyte adhesion to the vascular endothelium (Fig. 7a-b) without reducing ICAM-1 expression (Fig. 7c-d).

## Discussion

Far from being an inert vascular lining, ECs form a metabolically active and specialized interface between blood and underlying tissues. In response to an inflammatory stimulus, ECs undergo activation, which is classically defined by increased interaction with blood leukocytes. EC dysfunction plays a central role in peripheral vascular disease, stroke, heart disease, diabetes, insulin resistance, chronic kidney failure, tumor growth, metastasis, venous thrombosis, and severe viral infectious diseases [38]. The richly vascularized human brain contains ECs that form the nearly impenetrable BBB, which regulates the flux of substances in and out of the CNS. The contribution of cerebrovascular dysfunction in dementia and cognitive decline is well acknowledged [39, 40], yet pathways that mediate cerebrovascular EC dysfunction and protection are not well understood.

Using classical PMBC adhesion assays, we demonstrate that Aβ can act as an inflammatory stimulus to increase PBMC adhesion to brain microvascular ECs that can be suppressed by HDL. Our results show that the ability of HDL to attenuate Aβ-induced PBMC adhesion to ECs is independent of Aβ structure, NO production, miR-223 and, unexpectedly, of ICAM-1 and VCAM-1 expression. Rather, HDL requires SR-BI to suppress Aβ-mediated PBMC adhesion to ECs, which can also be attenuated by inhibiting internalization of Aβ by blocking RAGE or LRP1, or by inhibiting Aβ-HSPG interactions. We also show that HDL can also prevent Aβ fibrillization, suggesting that HDL may also help to maintain Aβ solubility as it transits across the BBB. However, as this function of HDL is relatively slow, it is unlikely to account for HDL’s ability to suppress Aβ-mediated PBMC adhesion to ECs. Our observations are consistent with a previous study that demonstrated that deficiency of SR-BI increases cerebrovascular amyloid deposition in the J20 mouse model of AD [41].

As ICAM-1 levels are clearly elevated in AD cortex, and previous studies have reported induced adhesion molecule expression after Aβ stimulation [42], we were surprised to observe that Aβ had negligible effects on adhesion molecule expression and kinase activation under our experimental conditions, whereas TNF-α robustly induces ICAM-1 and VCAM-1 expression as well as NFκB, MAPK/ERK, and SAPK/JNK signalling. The cellular processes triggered by internalized Aβ that promote PBMC adhesion remain to be determined.

In cultured aortic ECs, HDL reduces adhesion molecule expression and subsequent monocyte binding via a mechanism involving SR-BI signalling through eNOS, which requires the S1P receptor [25]. Our observations that HDL’s ability to suppress Aβ‑ and TNF-α-mediated PBMC adhesion to hCMEC/D3 is independent of NO production and S1P activity suggests that brain ECs may have pathways distinct from ECs derived from arterial sources, at least under the culture conditions used here. Reduced eNOS expression in the AD brain has been hypothesized to increase Aβ deposition and promote production of reactive oxygen species production, which reduces vasomotor regulation of penetrating arterioles [43, 44]. HDL clearly induces NO production in hCMEC/D3 and HUVEC, and although NO production is not required to protect hCMEC/D3 from Aβ- or TNF-α mediated PBMC adhesion per se, HDL-stimulated NO production may provide beneficial effects on other cerebrovascular EC functions.

A wealth of studies have explored in vitro associations of Aβ with lipoprotein components, primarily focussing on apoE as genetic variants of apoE have established effects on Aβ metabolism in AD [45]. As Aβ is a hydrophobic peptide, it is not surprising that it associates with lipoprotein particles, and also to amphipathic apolipoproteins. Elucidating the structure-function relationships of Aβ with lipoproteins remains an active area of research. While Aβ might associate with plasma lipoproteins [46], the effect of HDL on Aβ structure remains poorly understood. Interestingly, HDL constituents on their own have been reported to both interact with Aβ and diminish its toxic effects. Specifically, small liposomes (<50 μm) accelerate Aβ40 fibrillization and the amounts of amorphous aggregates become larger as liposome increase (>50 μm) [47]. By contrast, nanoliposomes (100 nm) prevent Aβ42 fibrillization and reduces Aβ42-induced EC dysfunctions [48]. In addition, lipid-free apoA-I induces Aβ aggregation and generation of amorphous complexes, both of which reducing amyloid toxicity [22]. Our results clearly show that mature HDL delays Aβ fibrillization in a cell-independent assay with substantially slower kinetics than HDL’s ability to attenuate Aβ-induced PBMC adhesion to ECs. As HDL also reduces Aβ-induced PBMC adhesion to ECs independent of monomeric vs. oligomeric input structures, we believe that HDL’s ability to delay Aβ fibrillization is a distinct function compared to reduce Aβ-inflammation, which relies exclusively to a cellular process involving SR-BI.

As traditional static monotypic cell cultures do not reproduce the physiological complexity of the native vascular bed, we also used a 3D model of the human vasculature in our studies. These engineered vessels mimic a native vessel with a luminal monolayer of human ECs surrounded by several layers of human SMCs, through which human mononuclear cells can be circulated under flow conditions. This model has been previously used to study atherosclerosis in peripheral arteries [17]. Here, we injected Aβ on the anteluminal side to mimic brain-produced Aβ and circulated HDL and monocytes through the lumen. We confirmed that Aβ induces monocyte adhesion to ECs under native-like flow conditions in engineered human vessels, which can be suppressed by HDL through a mechanism that requires SR-BI and that is independent of changes in ICAM-1 levels.

## Conclusion

Numerous studies show that HDL has several properties that are associated with improved vascular function [49]. While some lines of evidence also suggest a beneficial role for HDL in protecting from cognitive decline, the nature of this association remains largely unknown. Whereas lipid-free apoA-I can be transported into the CNS [50], there is thus far no evidence that mature HDL might cross the BBB, supporting the view that HDL might act predominately from the blood compartment and would therefore primarily affect ECs. Our data suggest at least three mechanisms by which HDL could have beneficial effects on cerebral vessels. First, HDL prevents Aβ-induced PBMC adhesion to brain microvascular ECs through a mechanism that requires SR-BI and suppresses Aβ uptake. The cellular pathways by which internalized Aβ promotes PBMC adhesion to ECs remains to be defined, and appears to be distinct from those induced by classical inflammatory stimuli. Second, similar to its effects in peripheral EC, HDL induces NO secretion in hCMEC/D3. Although we ruled out a role for eNOS in Aβ-induced PBMC adhesion, HDL-stimulated NO production may still help to attenuate vasomotor dysfunction as observed in the aging brain [44]. Third, although HDL can protect hCMEC/D3 from PBMC adhesion independent of Aβ structure, the ability of HDL to maintain Aβ in a soluble state may facilitate efficient Aβ clearance out of the brain.

## Additional files

Additional file 1: Aβ induced dose dependent PBMC adhesion and HDL attenuate dose dependent Aβ-induced PBMC adhesion to hCMEC/D3. In all conditions, hCMEC/D3 were stimulated with 0–1 μM Aβ40 (light grey) or Aβ42 (dark grey) monomers for 3 h. Fluorescently labelled PBMC were allowed to adhere to (**a**) Aβ40- or (**b**) Aβ42- stimulated cells for 3 additional hours. Cells were washed, fixed, and imaged to count adhered PBMC. hCMEC/D3 were primed with increasing doses (25–400 μg/mL) of HDL for 2 h and stimulated with 0.1 μM (**c**) Aβ40 (light grey) or (**d**) Aβ42 (dark grey) for 3 h. Fluorescently labelled PBMC were allowed to adhere to stimulated cells for 3 h followed by washing, fixation, imaging, and counting. Graphs represent mean ± SD of adhered PBMC relative to vehicle control from at least 3 independent trials where * *p* < 0.05, **p < 0.01, ***p < 0.001 versus vehicle, § *p* < 0.05, §§ *p* < 0.01 versus Aβ (PDF 422 kb)

Additional file 2 HDL suppression of Aβ-induced inflammation is independent of eNOS and S1P in HUVEC. (**a**) L-NAME and (**e**) VPC23019 potency was tested by measuring intracellular NO production in HUVEC after incubating with 100 μg/mL HDL and 1 μM DAF-2 for 6 h. Fluorescence was measured at 485 nm. (**b-d, f-h**) In all conditions, HUVEC or hCMEC/D3 were stimulated with 0.1 μM **monomeric** Aβ40 or Aβ42 or 1 ng/mL of TNF-α for 3 h prior to measuring PBMC adherence. HUVEC were pre-treated for 1 h with (**b-d**) the eNOS inhibitor L-NAME or (**f-h**) the S1P1 and S1P3 inhibitor VPC23019 followed by 100 μg/mL HDL for 2 h. (**i**) hCMEC/D3 were pre-treated with 100 μg/mL of HDL for 2 h before simulating with Aβ40 or Aβ42. Total cellular expression of Annexin-1 (Anx1) was analysed by immunoblotting and compared to GAPDH. Graphs represent means ± SD from at least 3 independent trials. * *p* < 0.05, **p < 0.01, ***p < 0.001, ****p < 0.0001 versus vehicle, § *p* < 0.05, §§ *p* < 0.01, §§§*p* < 0.001, §§§§ *p* < 0.0001 versus Aβ or TNF-α (PDF 1059 kb)

Additional file 3: HDL does not signal through miR-223 to reduce Aβ-induced inflammation in hCMEC/D3. (**a-c**) hCMEC/D3 were pre-treated with 100 μg/mL of HDL as described in Fig. 2 with or without 10 nM of miR-223 mimetic nucleotides or (**d-f**) in the absence or presence of a specific miR223 inhibitor for 2 h before stimulation with (**a,d**) Aβ40, (**b,e**) Aβ42 or (**c, f**) TNF-α before testing PBMC adherence to ECs. (**g**) Intracellular levels of mature miR-223 in hCMEC/D3 were quantified by real-time PCR and normalized to U6 after a 5 h treatment with 100 μg/mL of HDL. Graphs represent means ± SD of adhered PBMC relative to vehicle treated cells for at least 5 independent trials. *p < 0.05, **p < 0.01, ***p < 0.001 * *p* < 0.05, **p < 0.01, ***p < 0.001 versus vehicle, § *p* < 0.05, §§ *p* < 0.01, §§§*p* < 0.001 versus Aβ or TNF-α (PDF 464 kb)

Additional file 4: Demographic data, Aβ40 and Aβ42, and adhesion molecule quantification in Alzheimer’s disease patients and non-cognitive impaired controls (PDF 431 kb)

Additional file 5: Cortical ICAM-1 expression is increased in AD. Cryopreserved cortex and cerebellum of AD or NCI patients were cut at 20 μm. After PFA fixation sections were washed with PBS and stained against (**a**) ICAM-I or (**b**) VCAM-1 and CD31 as a vascular marker and imaged using an inverted fluorescent microscope. Arrow demonstrates colocalization of ICAM-1 and CD31. Bar represents 50 μm (PDF 62676 kb)

Additional file 6: Adhesion molecules are enhanced by TNF-α but not Aβ.(**a-c**) hCMEC/D3 were primed with 100 μg/mL HDL for 2 h followed by stimulation with 1 ng/mL TNF-α for 3 h. Cell lysates were prepared in RIPA and protein levels of (**a**) ICAM-1 and (**b**) VCAM-1 were measured by denaturing immunoblotting **(c)**. (**d-g**) hCMEC/D3 were stimulated with monomeric (**d,f**) Aβ40 or (**e,**g) Aβ42 at the indicated concentrations and (**d,e**) *ICAM-1* and (**f,g**) *VCAM-1* mRNA levels were measured by real-time PCR. (**h-j**) Following Aβ stimulation, cell surface proteins were biotinylated and isolated by immunoprecipitation. Protein levels of cell surface and total (**h)** ICAM-1 and (**i**) VCAM-1 were measured by denaturing immunoblotting **(j)**. (**k-m**) hCMEC/D3 were pre-treated with 100 μg/mL of HDL for 2 h followed by stimulation with Aβ. After 3 h total and cell surface ICAM-1 expression were measured as above. Graphs represent means ± SD from at least 3 independent trials. ***p < 0.001 versus vehicle, § *p* < 0.05, versus Aβ or TNF-α (PDF 2282 kb)

Additional file 7: Aβ does not activate phosphorylation of multifunctional serine/threonine protein kinases. hCMEC/D3 were stimulated with 0.1 mM of monomeric Aβ or 1 ng/mL of TNF-αfor 15 min before lysing cells in RIPA containing phosphostop. Phosphorylation of (**a-b**) p65, (**c**) STAT3, (**d**) Akt, (**e**) SAPK/JNK and (**f**) p42/44 MAPK were analysed by immunoblotting and compared to respective total p65, STAT3, Akt, SAPK/JNK and p42/44 MAPK respectively. (**b**) Nuclear translocation of p65 was analysed by immunofluorescence 15 min after Aβ stimulation. Graphs represent means ± SD relative to vehicle treated cells in 4 trials. *p < 0.05, **p < 0.01 (PDF 9645 kb)

Additional file 8: HDL treatment does not alter LRP1 or RAGE protein levels in hCMEC/D3. (**a-c**) hCMEC/D3 were treated with HDL (0–400 μg/mL) for 5 h before lysing in RIPA. (**a**) LRP1 and (**b**) RAGE protein levels were quantified by immunoblotting (**c**). Graphs represent means ± SD relative to vehicle treated cells in 3 trials (PDF 1114 kb)

## Abbreviations

AD: Alzheimer Disease
Aβ: Beta-amyloid peptide
BLT-1: Block lipid transport 1
CAA: Cerebral amyloid angiopathy
CNS: Central nervous system
EBM-2: Endothelial growth basal medium
EC: Endothelial cell
eNOS: Endothelial nitric oxide synthase
FBS: Fetal bovine serum
FITC: Fluorescein isothiocyanate
hBMEC: Human brain microvascular endothelial cell
hCMEC/D3: Human cortical microvascular endothelial cell line D3
HDL: High density lipoprotein
hSMC: Human smooth muscle cell
HSPG: Heparan sulphate proteoglycan
ICAM-1: Inducible cellular adhesion molecule-1
L-NAME: L–NG-nitroarginne methyl ester
LRP1: Low density lipoprotein receptor related protein-1
NO: Nitric oxide
PBMC: Peripheral blood mononuclear cell
RAGE: Receptor of advanced glycation endproducts
SR-BI: Scavenger receptor BI
VCAM-1: Vascular cell adhesion molecule −1
