# Supplementary figures and images for: High-density lipoproteins suppress Aβ-induced PBMC adhesion to human endothelial cells in bioengineered vessels and in monoculture
Source: Mol Neurodegener. 2017 Aug 22;12:60. doi: 10.1186/s13024-017-0201-0 (PMC5568306; doi:10.1186/s13024-017-0201-0)

Additional File 1

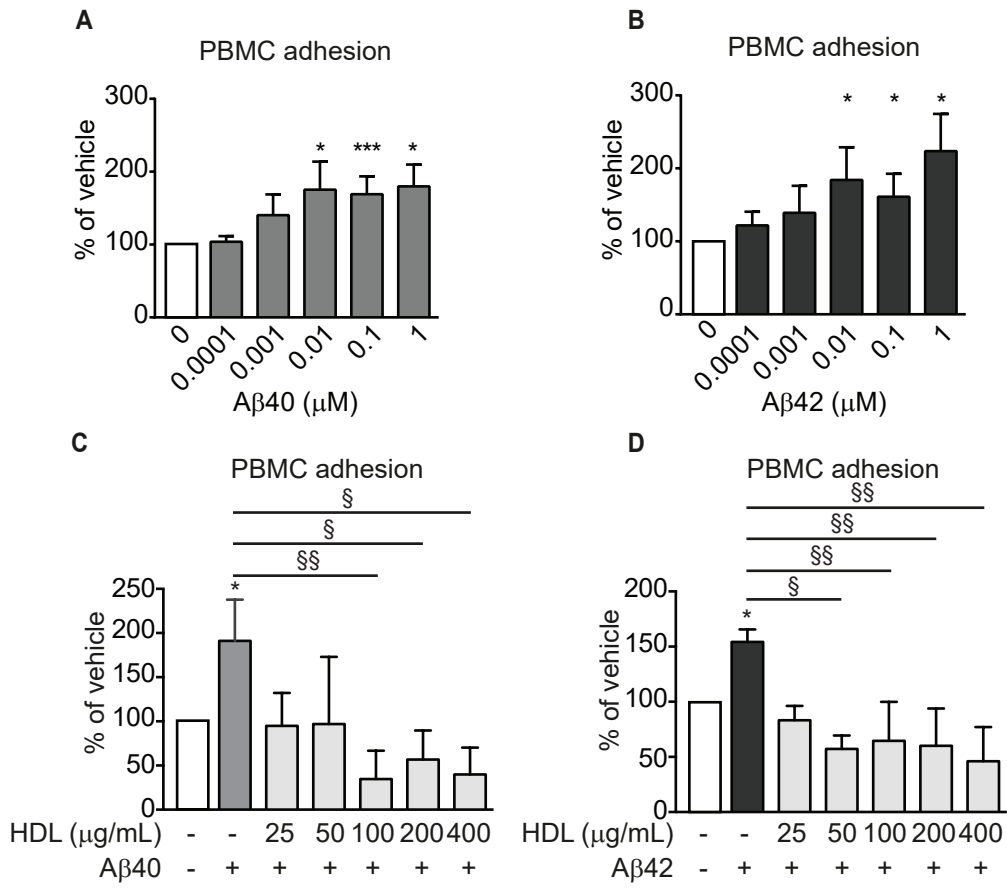

Supplement: Supplementary file 1 — Aβ induced dose dependent PBMC adhesion and HDL attenuate dose dependent Aβ-induced PBMC adhesion to hCMEC/D3. In all conditions, hCMEC/D3 were stimulated with 0–1 μM Aβ40 (light grey) or Aβ42 (dark grey) monomers for 3 h. Fluorescently labelled PBMC were allowed to adhere to (a) Aβ40- or (b) Aβ42- stimulated cells for 3 additional hours. Cells were washed, fixed, and imaged to count adhered PBMC. hCMEC/D3 were primed with increasing doses (25–400 μg/mL) of HDL for 2 h and stimulated with 0.1 μM (c) Aβ40 (light grey) or (d) Aβ42 (dark grey) for 3 h. Fluorescently labelled PBMC were allowed to adhere to stimulated cells for 3 h followed by washing, fixation, imaging, and counting. Graphs represent mean ± SD of adhered PBMC relative to vehicle control from at least 3 independent trials where * p < 0.05, **p < 0.01, ***p < 0.001 versus vehicle, § p < 0.05, §§ p < 0.01 versus Aβ (PDF 422 kb) [file 13024_2017_201_MOESM1_ESM.pdf]

**Additional File 2**

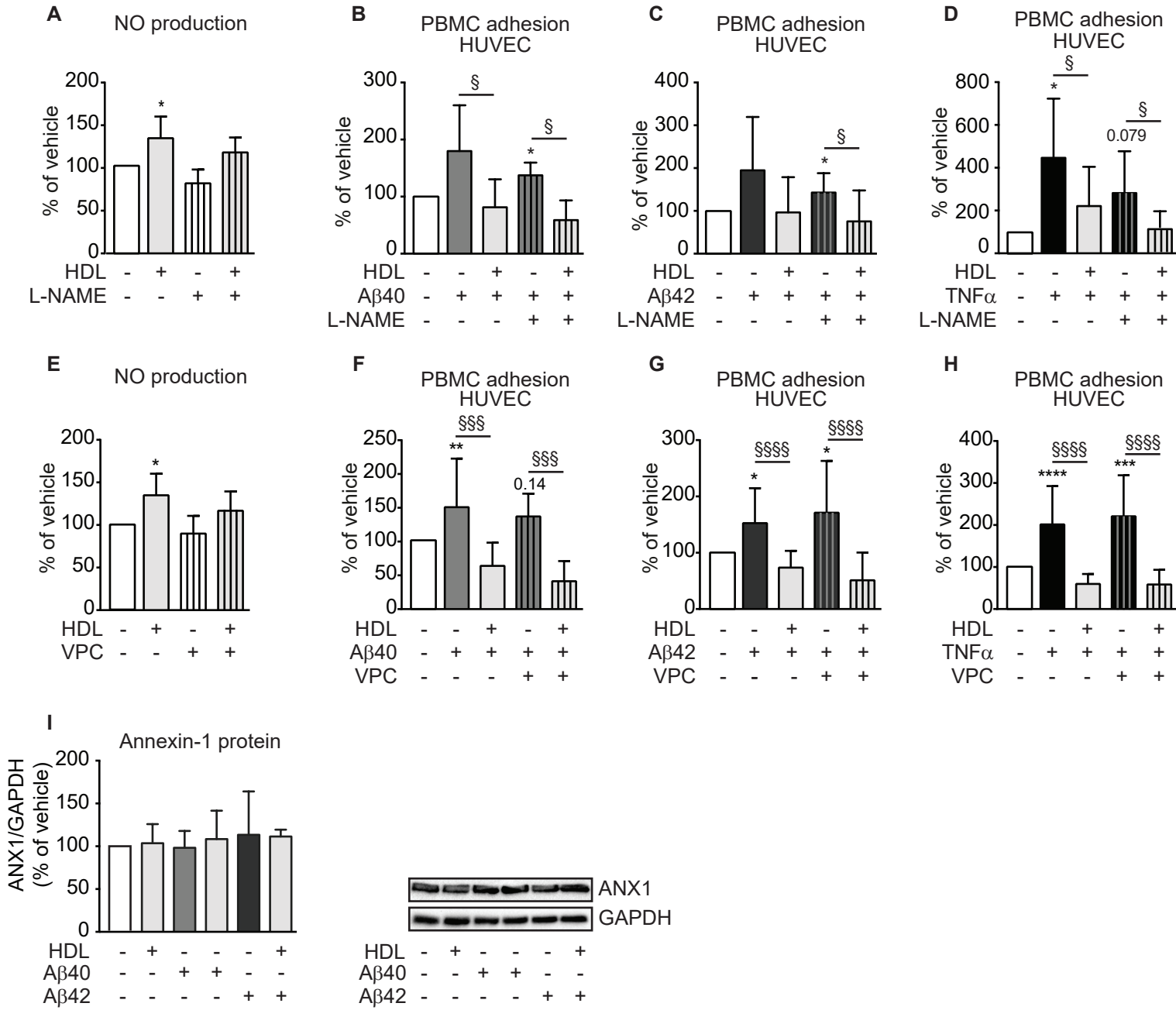

Supplement: Supplementary file 2 — HDL suppression of Aβ-induced inflammation is independent of eNOS and S1P in HUVEC. (a) L-NAME and (e) VPC23019 potency was tested by measuring intracellular NO production in HUVEC after incubating with 100 μg/mL HDL and 1 μM DAF-2 for 6 h. Fluorescence was measured at 485 nm. (b-d, f-h) In all conditions, HUVEC or hCMEC/D3 were stimulated with 0.1 μM monomeric Aβ40 or Aβ42 or 1 ng/mL of TNF-α for 3 h prior to measuring PBMC adherence. HUVEC were pre-treated for 1 h with (b-d) the eNOS inhibitor L-NAME or (f-h) the S1P1 and S1P3 inhibitor VPC23019 followed by 100 μg/mL HDL for 2 h. (i) hCMEC/D3 were pre-treated with 100 μg/mL of HDL for 2 h before simulating with Aβ40 or Aβ42. Total cellular expression of Annexin-1 (Anx1) was analysed by immunoblotting and compared to GAPDH. Graphs represent means ± SD from at least 3 independent trials. * p < 0.05, **p < 0.01, ***p < 0.001, ****p < 0.0001 versus vehicle, § p < 0.05, §§ p < 0.01, §§§p < 0.001, §§§§ p < 0.0001 versus Aβ or TNF-α (PDF 1059 kb) [file 13024_2017_201_MOESM2_ESM.pdf]

Additional File 3

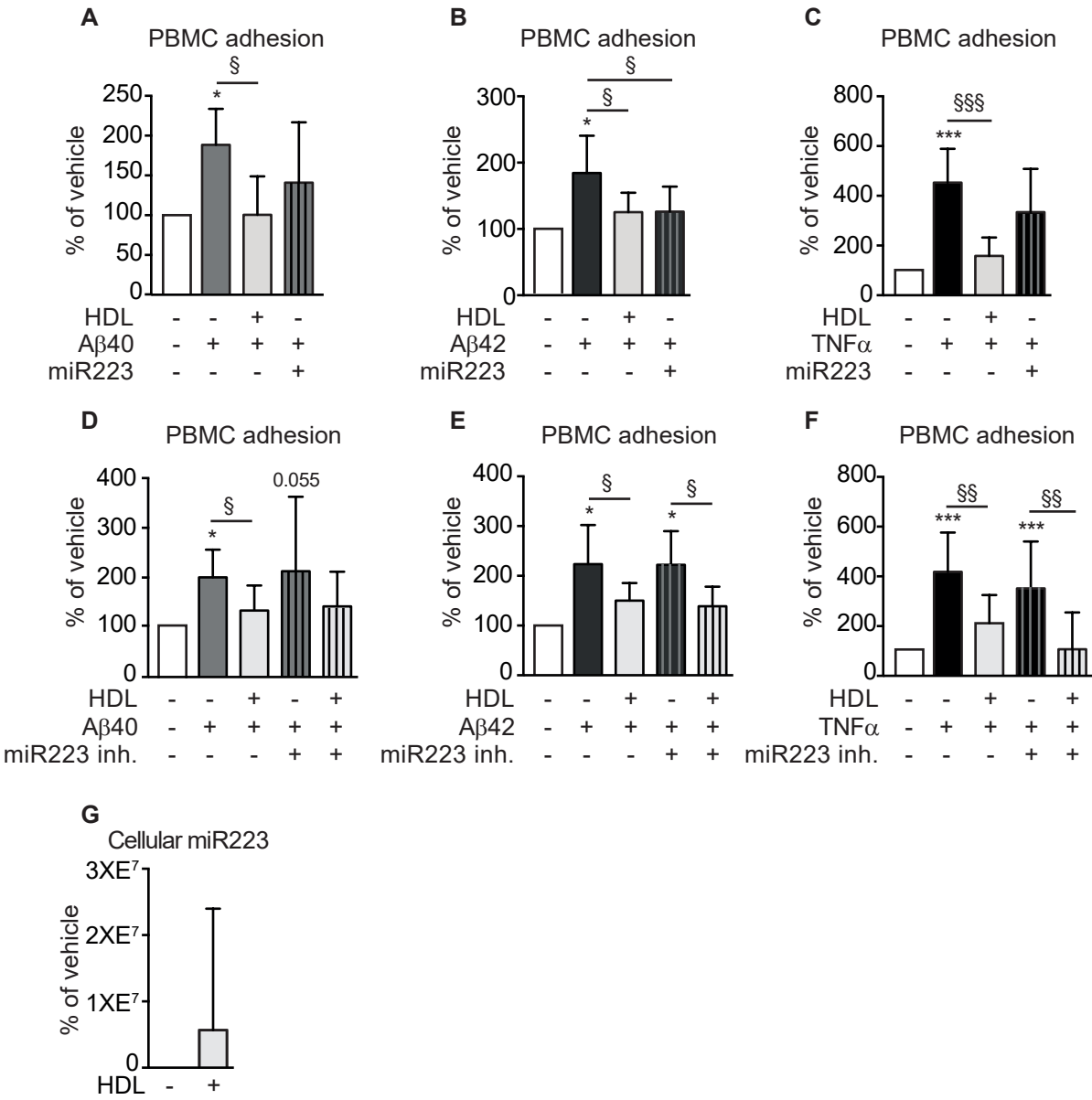

Supplement: Supplementary file 3 — HDL does not signal through miR-223 to reduce Aβ-induced inflammation in hCMEC/D3. (a-c) hCMEC/D3 were pre-treated with 100 μg/mL of HDL as described in Fig. 2 with or without 10 nM of miR-223 mimetic nucleotides or (d-f) in the absence or presence of a specific miR223 inhibitor for 2 h before stimulation with (a,d) Aβ40, (b,e) Aβ42 or (c, f) TNF-α before testing PBMC adherence to ECs. (g) Intracellular levels of mature miR-223 in hCMEC/D3 were quantified by real-time PCR and normalized to U6 after a 5 h treatment with 100 μg/mL of HDL. Graphs represent means ± SD of adhered PBMC relative to vehicle treated cells for at least 5 independent trials. *p < 0.05, **p < 0.01, ***p < 0.001 * p < 0.05, **p < 0.01, ***p < 0.001 versus vehicle, § p < 0.05, §§ p < 0.01, §§§p < 0.001 versus Aβ or TNF-α (PDF 464 kb) [file 13024_2017_201_MOESM3_ESM.pdf]

Additional File 5

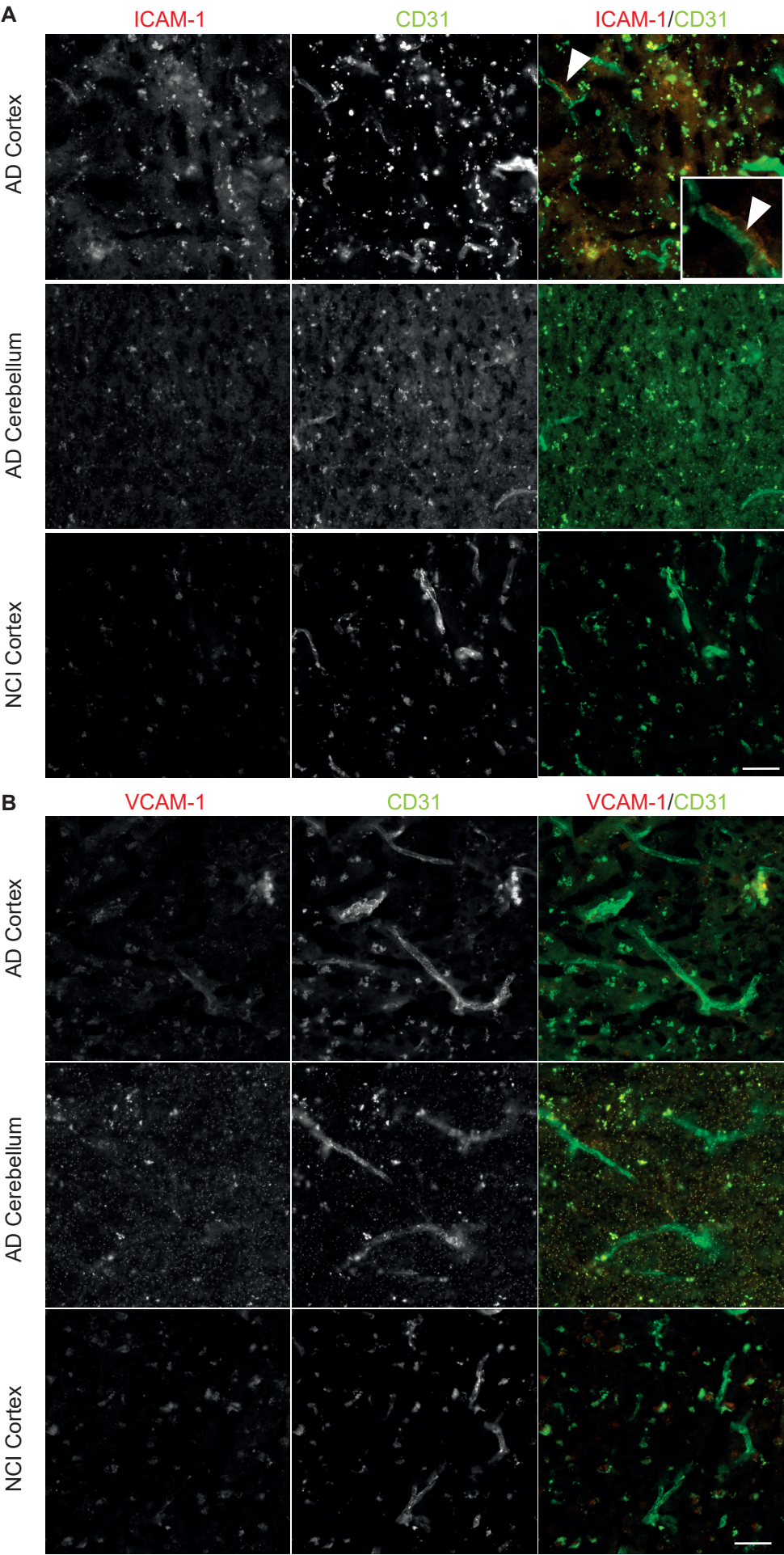

Supplement: Supplementary file 5 — Cortical ICAM-1 expression is increased in AD. Cryopreserved cortex and cerebellum of AD or NCI patients were cut at 20 μm. After PFA fixation sections were washed with PBS and stained against (a) ICAM-I or (b) VCAM-1 and CD31 as a vascular marker and imaged using an inverted fluorescent microscope. Arrow demonstrates colocalization of ICAM-1 and CD31. Bar represents 50 μm (PDF 62676 kb) [file 13024_2017_201_MOESM5_ESM.pdf]

Additional File 6

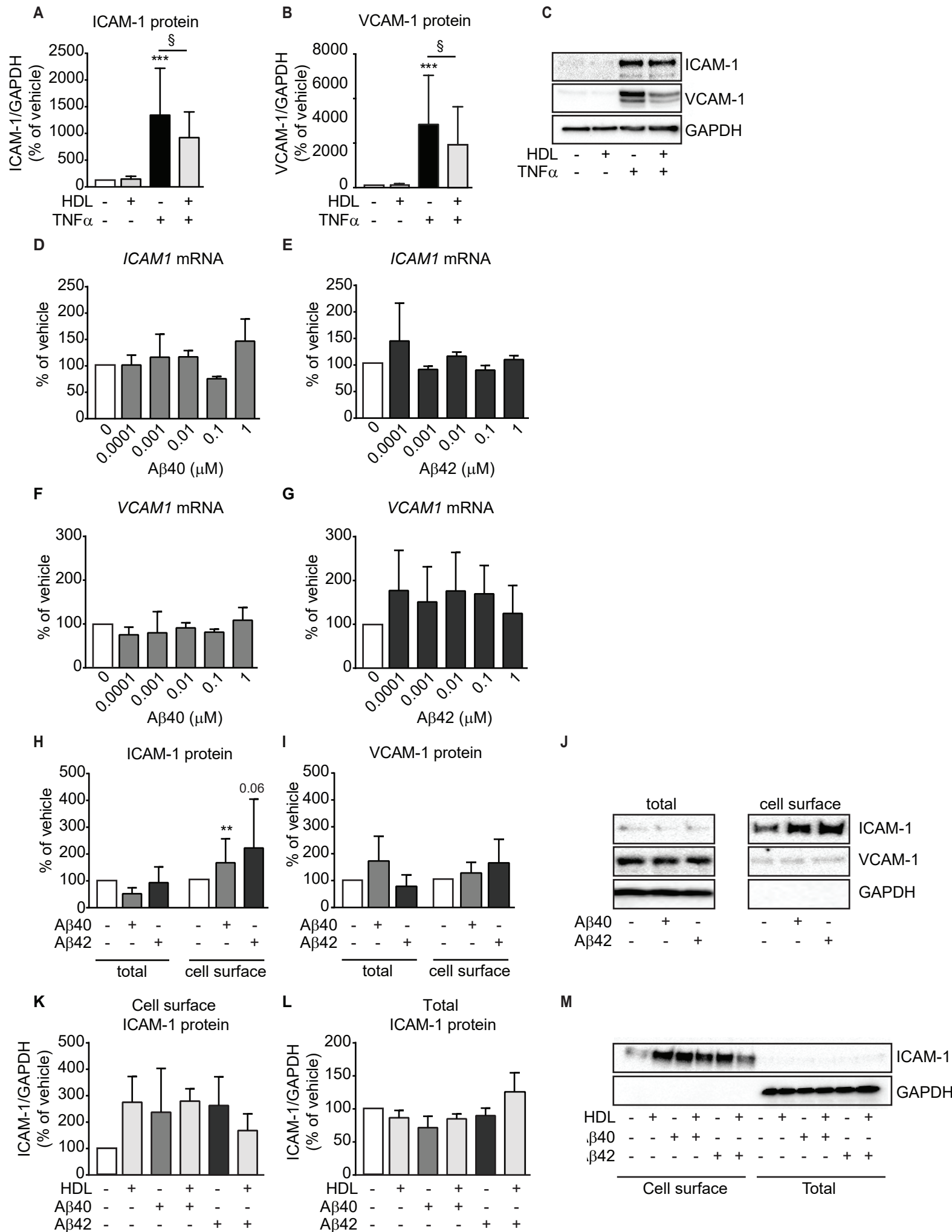

Supplement: Supplementary file 6 — Adhesion molecules are enhanced by TNF-α but not Aβ.(a-c) hCMEC/D3 were primed with 100 μg/mL HDL for 2 h followed by stimulation with 1 ng/mL TNF-α for 3 h. Cell lysates were prepared in RIPA and protein levels of (a) ICAM-1 and (b) VCAM-1 were measured by denaturing immunoblotting (c). (d-g) hCMEC/D3 were stimulated with monomeric (d,f) Aβ40 or (e,g) Aβ42 at the indicated concentrations and (d,e) ICAM-1 and (f,g) VCAM-1 mRNA levels were measured by real-time PCR. (h-j) Following Aβ stimulation, cell surface proteins were biotinylated and isolated by immunoprecipitation. Protein levels of cell surface and total (h) ICAM-1 and (i) VCAM-1 were measured by denaturing immunoblotting (j). (k-m) hCMEC/D3 were pre-treated with 100 μg/mL of HDL for 2 h followed by stimulation with Aβ. After 3 h total and cell surface ICAM-1 expression were measured as above. Graphs represent means ± SD from at least 3 independent trials. ***p < 0.001 versus vehicle, § p < 0.05, versus Aβ or TNF-α (PDF 2282 kb) [file 13024_2017_201_MOESM6_ESM.pdf]

Additional File 7

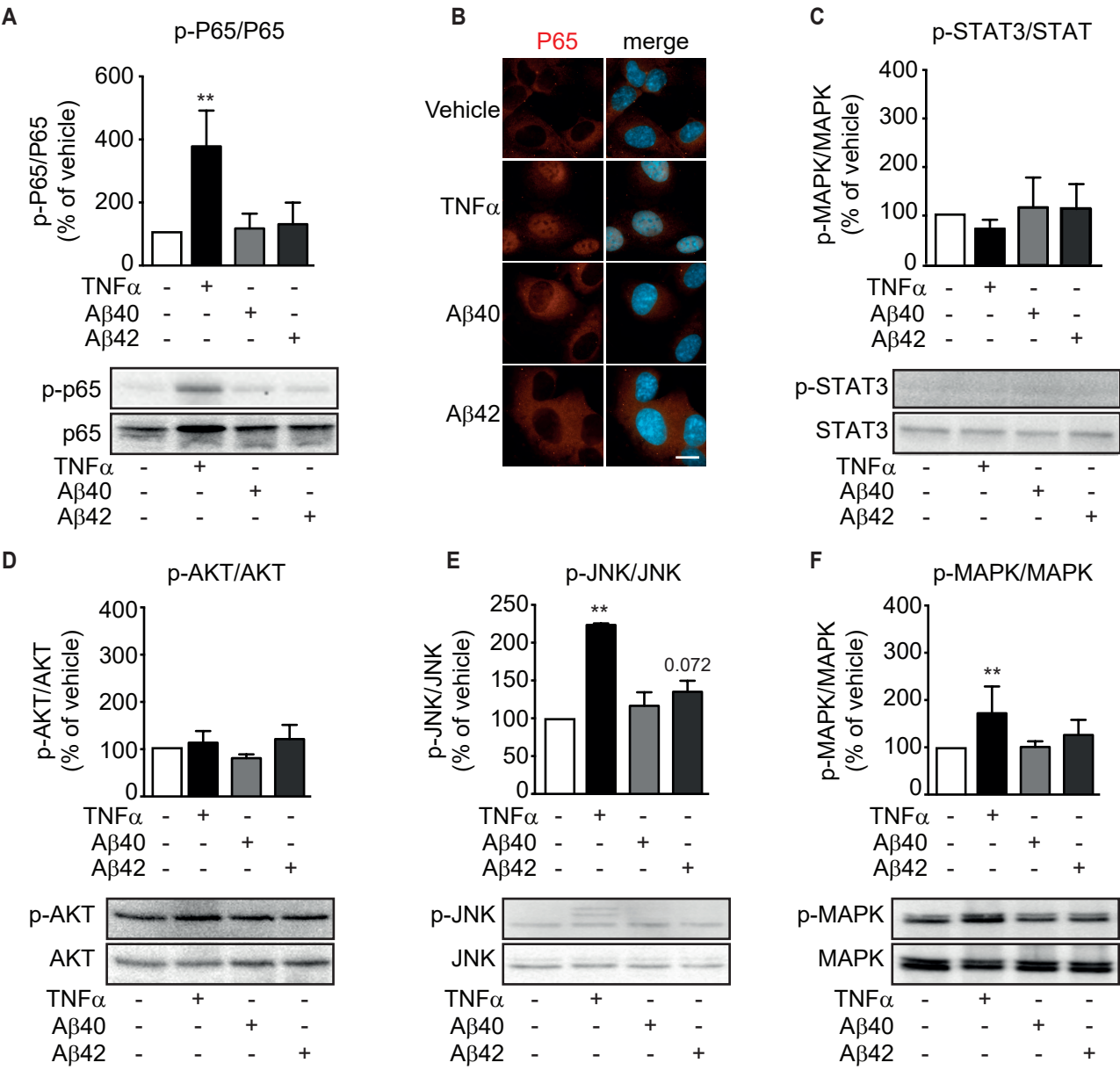

Supplement: Supplementary file 7 — Aβ does not activate phosphorylation of multifunctional serine/threonine protein kinases. hCMEC/D3 were stimulated with 0.1 mM of monomeric Aβ or 1 ng/mL of TNF-αfor 15 min before lysing cells in RIPA containing phosphostop. Phosphorylation of (a-b) p65, (c) STAT3, (d) Akt, (e) SAPK/JNK and (f) p42/44 MAPK were analysed by immunoblotting and compared to respective total p65, STAT3, Akt, SAPK/JNK and p42/44 MAPK respectively. (b) Nuclear translocation of p65 was analysed by immunofluorescence 15 min after Aβ stimulation. Graphs represent means ± SD relative to vehicle treated cells in 4 trials. *p < 0.05, **p < 0.01 (PDF 9645 kb) [file 13024_2017_201_MOESM7_ESM.pdf]

Additional File 8

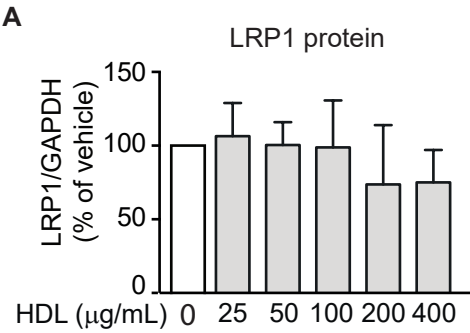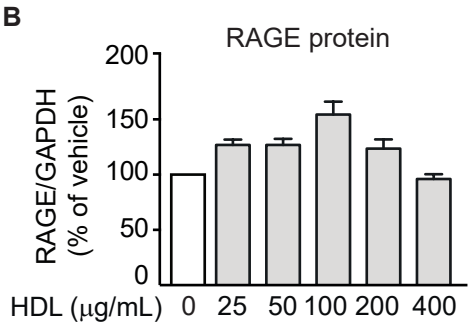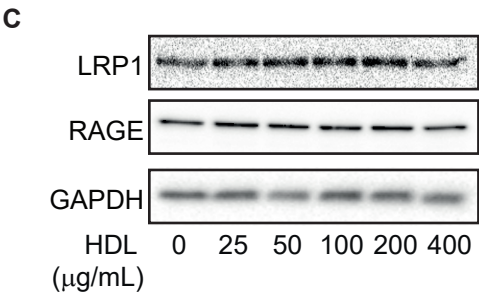

Supplement: Supplementary file 8 — HDL treatment does not alter LRP1 or RAGE protein levels in hCMEC/D3. (a-c) hCMEC/D3 were treated with HDL (0–400 μg/mL) for 5 h before lysing in RIPA. (a) LRP1 and (b) RAGE protein levels were quantified by immunoblotting (c). Graphs represent means ± SD relative to vehicle treated cells in 3 trials (PDF 1114 kb) [file 13024_2017_201_MOESM8_ESM.pdf]
