# Supplementary material for: High-density lipoproteins suppress Aβ-induced PBMC adhesion to human endothelial cells in bioengineered vessels and in monoculture
Source: Mol Neurodegener. 2017 Aug 22;12:60. doi: 10.1186/s13024-017-0201-0 (PMC5568306; doi:10.1186/s13024-017-0201-0)
Supplement: Supplementary file 4 — Demographic data, Aβ40 and Aβ42, and adhesion molecule quantification in Alzheimer’s disease patients and non-cognitive impaired controls (PDF 431 kb) [file 13024_2017_201_MOESM4_ESM.pdf]

Additional File 4

Group (n)	NCI (5)	AD (5)	
Brain regions	Cortex	Cortex	Cerebellum
Average age (mean±SD)	62±3.77	67±9.67	
Sex (% male)	100	40	
Stage	Control	Braak IV	
Aβ40 (ng/mg)	0.87±0.36	1.42±0.22*	0.79± 0.13
Aβ42 (ng/mg)	0.02±0.03	1.58±0.91**	0.13± 0.2
ICAM (pg/mg)	3.98±1.78	24.88±15.19**	4.13± 1.22
VCAM (pg/mg)	23.08±8.21	25.35±8.82	22.80± 2.19
